# Cyclovirobuxine D protects against diabetic cardiomyopathy by activating Nrf2-mediated antioxidant responses

**DOI:** 10.1038/s41598-020-63498-3

**Published:** 2020-04-14

**Authors:** Zhaohui Jiang, Lingyun Fu, Yini Xu, Xiaoxia Hu, Hong Yang, Yanyan Zhang, Hong Luo, Shiquan Gan, Ling Tao, Guiyou Liang, Xiangchun Shen

**Affiliations:** 1 The State Key Laboratory of Functions and Applications of Medicinal Plants, School of Basic Medical Sciences, Guizhou Medical University, University Town, Guian New District, 550025 Guizhou, China; 2 The Department of Pharmacology of Materia Medica (The high efficacy application of natural medicinal resources engineering center of Guizhou Province and The high educational key laboratory of Guizhou province for natural medicianl Pharmacology and Druggability), School of Pharmaceutical Sciences, Guizhou Medical University, University Town, Guian New District, 550025 Guizhou, China; 3 The key laboratory of optimal Utilizaiton of Natural Medicine Resources (The union key laboratory of Guiyang City-Guizhou Medical Univeristy), School of Pharmaceutical Sciences, Guizhou Medical University, University Town, Guian New District, 550025 Guizhou, China; 4 The key laboratory of Endemic and Ethnic diseases of Ministry of Education, Guizhou Medical University, 550004 Guizhou, China

**Keywords:** Cardiology, Pharmacology

## Abstract

Diabetic cardiomyopathy (DCM) is the principal cause of death in people with diabetes. However, there is currently no effective strategy to prevent the development of DCM. Although cyclovirobuxine D (CVB-D) has been widely used to treat multiple cardiovascular diseases, the possible beneficial effects of CVB-D on DCM remained unknown. The present aim was to explore the potential effects and underlying mechanisms of CVB-D on DCM. We explored the effects of CVB-D in DCM by using high fat high sucrose diet and streptozotocin-induced rat DCM model. Cardiac function and survival in rats with DCM were improved via the amelioration of oxidative damage after CVB-D treatment. Our data also demonstrated that pre-treatment with CVB-D exerted a remarkable cytoprotective effect against high glucose -or H_2_O_2_ -induced neonatal rat cardiomyocyte damage via the suppression of reactive oxygen species accumulation and restoration of mitochondrial membrane potential; this effect was associated with promotion of Nrf2 nuclear translocation and its downstream antioxidative stress signals (NQO-1, Prdx1). Overall, the present data has provided the first evidence that CVB-D has potential therapeutic in DCM, mainly by activation of the Nrf2 signalling pathway to suppress oxidative stress. Our findings also have positive implications on the novel promising clinical applications of CVB-D.

## Introduction

Diabetic cardiomyopathy (DCM) is usually characterised by cardiac structure and functional disorders in individuals with diabetes independent of hypertension or ischemic coronary artery disease^1–3^. Although it is the principal cause of death in patients with diabetes, no effective strategies currently exist to prevent the progression of DCM^4^. The pathogenesis of DCM involves in many factors such as oxidative stress, chronic low-grade inflammation^5^, autophagy^6^, and pyroptosis^7^, etc. The accumulated evidences confirm that cardiomyocyte injuries induced by oxidative stress are the predominant contributors to the pathophysiological process of DCM^8^. Reactive oxygen species (ROS) overproduction induced by hyperglycaemia, free fatty acids, and glycosylation end products results in myocardial structural damage and functional or metabolic disorders, which are considered to be the key pathological signal of DCM^9^. Therefore, amelioration of oxidative stress may be a therapeutic strategy to prevent the progression of DCM.

Nuclear factor (carotenoid-derived 2)-like 2 (Nrf2) is the major transcription factor in the cellular antioxidant response^10^. The expression and activity of Nrf2 are regulated by cullin 3-based ubiquitin E3 ligases, such as Kelch-like ECH-related protein 1 (Keap1). Upon exposure to various stress conditions, Nrf2 is uncoupled from keap1 and subsequently translocated to the nucleus, which activates downstream defence genes, such as NAD(P)H: quinone oxidoreductase 1 (NQO-1) and peroxide enzyme 1 (Prdx1)^11,12^. As oxidative stress is a common pathogenesis of metabolic diseases, some pharmacological activators of Nrf2, such as mitoquinone^13,14^, bardoxolone methyl^15^, and sulforaphane^16^, have shown beneficial therapeutic potential. However, off-target side effects also were observed because of the unselective activity of Nrf2^17–19^. Therefore, it is important for exploring the novel promising agent targeted Nrf2 agonist for the DCM.

Cyclovirobuxine D (CVB-D; molecular formula: C_26_H_46_N_2_O) is a triterpenoid alkaloid extracted from Caulis et Ramulus Buxi Sinicae (a traditional Chinese medicine). Plenty of data have suggested that CVB-D has widely applied to cardiovascular diseases, such as heart failure, coronary heart disease, and myocardial ischemia^20–22^. CVB-D has been demonstrated multiple pharmacological activities, including antioxidant, anti-inflammatory, and autophagy-regulating effects^23,24^. Moreover, CVB-D protected against cardiac hypertrophy via the inhibition of the p38 pathways^25^. However, it remains unclear if CVB-D could ameliorate oxidative stress via the activation of Nrf2 signalling pathways and thereby prevent the cardiac pathologic structural damage and function disorder caused by diabetes. At present, we investigate the beneficial effects and potential mechanism of CVB-D on DCM *in vivo* and *in vitro*.

## Results

### CVB-D improves cardiac function in DCM rats

**Figure 1:**
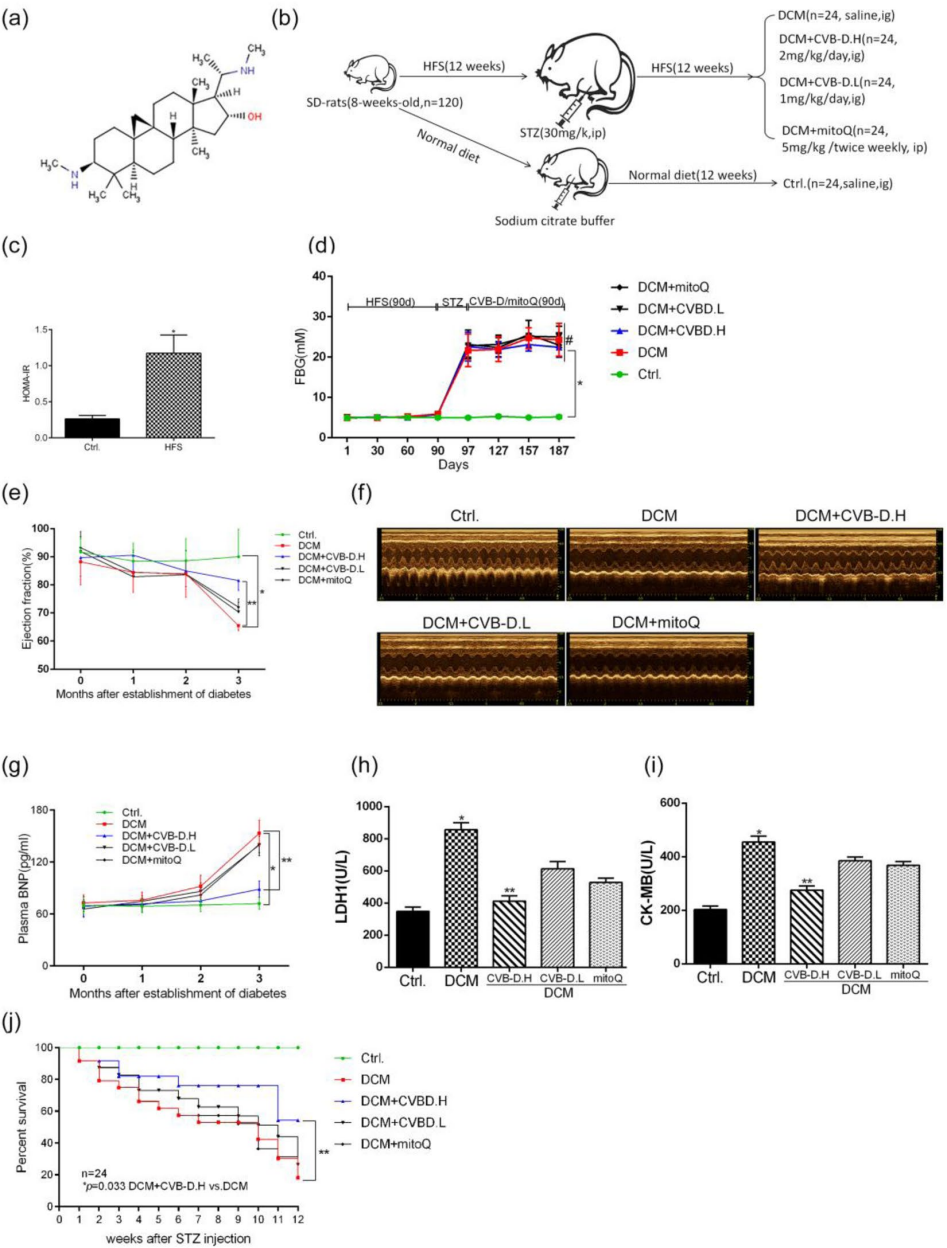
CVB-D ameliorates cardiac function in rats with DCM. (**a**) The chemical structure of CVB-D. (**b**)Experimental design. (**c**) Insulin resistance (HOMA-IR) obtained after 12 weeks of HFS. (**d**) FBG was monitored during the experiment. Ejection fractions (**e**) and serum BNP levels (**g**) were monitored at the end of every month after type 2 diabetes was successfully established. (**f**) Representative images of M-mode echocardiography after 12 weeks of treatment with CVB-D. Serum LDH1 (**h**) and CK-MB (**i**) levels obtained after treatment with CVB-D for 12 weeks. (**j**) Kaplan-Meier survival curves and Mantel-Cox log rank test were used to analysis the survival rates for rats from each group; *p* = 0.0033, CVB-D.H vs. M. n (Ctrl.) =24; n (DCM) =7; n (DCM + CVB-D.H) =16; n (DCM + CVB-D.L) =8; n(DCM + mitoQ)=9. Data were expressed as the mean ± SD. *p < 0.05 versus the control group; **p < 0.05 versus the model group; #*p* > 0.05 versus the model group.

The type 2 diabetes rat model was reproduced by the injection of small doses of STZ and feeding high fat high sucrose(HFS)-fodder. As shown in Fig. 1c, the rats showed insulin resistance after HFS feeding for 12 weeks. The levels of FBG were remarkably increased in the model group compared with the control group (Fig. 1d). Cardiac function parameters were measured by electrocardiograph and plasma BNP levels, and our data showed that the ejection fraction (EF) (Fig. 1e,f) and LVFS (Fig. S1a) of diabetic rats showed a downward trend, while BNP levels gradually increased (Fig. 1g). Administrating with CVB-D (2 mg/kg/day) for 12 weeks could ameliorate these pathological changes. The LDH1 and CK-MB activity as biomarkers in serum were evaluated cardiac injury, which was obviously increased in DCM, and was significantly inhibited treated with CVB-D (Fig. 1e–i). CVB-D significantly improved the survival rate of DCM rats (Fig. 1j). Compared with the control, the LV mass was increased in DCM, CVB-D has no significant effect on this change (Supplementary Fig. S1b) similar to LVPWd and LVPWs (Fig. S1c,d). CVB-D did not alleviate the FBG levels (Fig. 1d), which indicated CVB-D ameliorated cardiac function independent on blood glucose control. Collectively, these data suggested that the diabetic group had developed DCM, whereas CVB-D treatment alleviated cardiac dysfunction.

### CVB-D ameliorates oxidative stress in DCM heart

**Figure 2:**
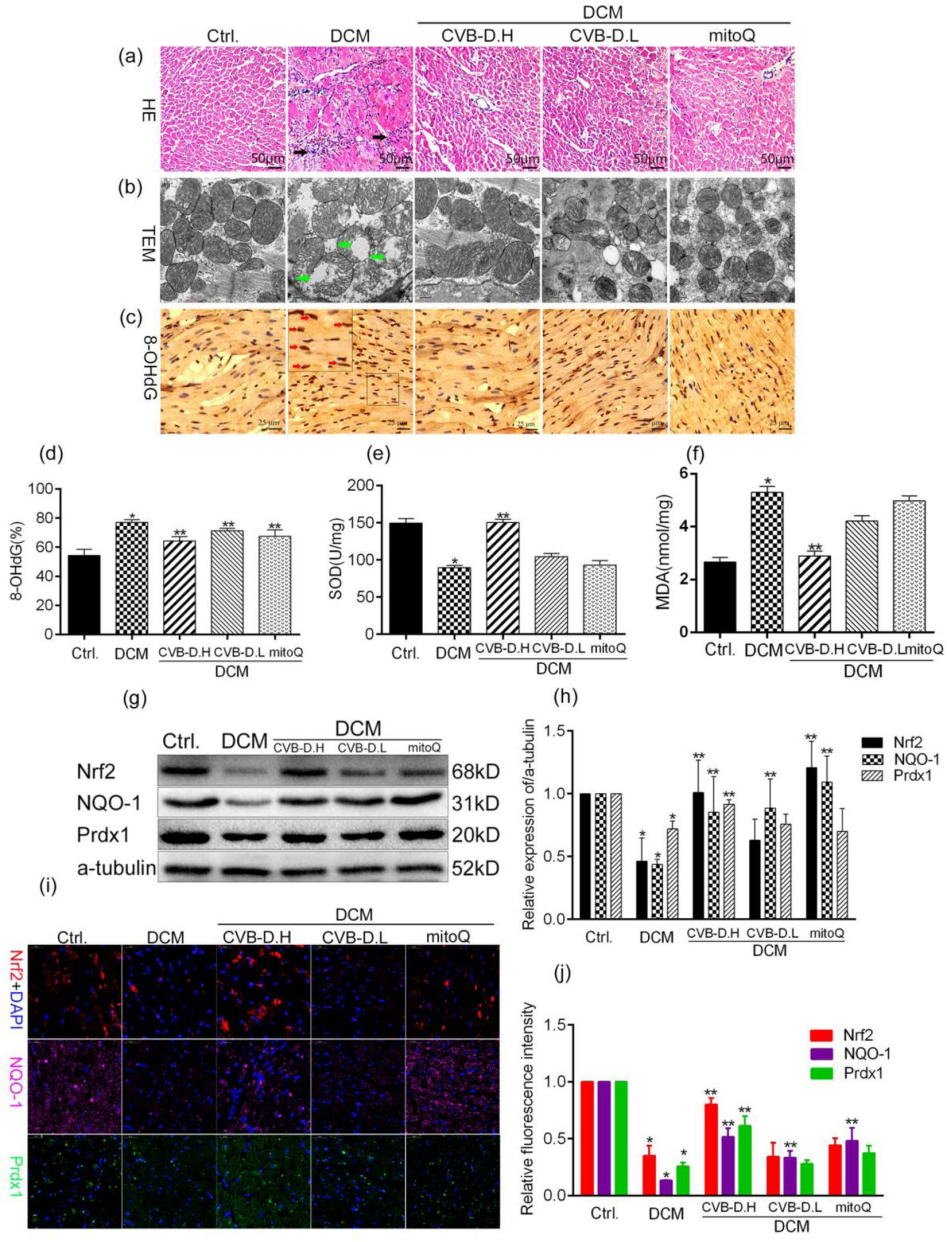
CVB-D ameliorates oxidative damage in the heart of rats with DCM. (**a**) Representative section HE staining of heart tissues (scale bar = 50 μm). (**b**)Representative transmission electron micrographs of mitochondria in heart tissues (scale bar = 200 μm). (**c,d**) Representative images of immunohistochemical staining for 8-OHdG (scale bar = 25 μm). (**e**) SOD activity. (**f**) MDA content. (**g**) Representative blots of Nrf2, NQO-1, Prdx-1 and a-tubulin, full–length blots are presented in Supplementary Fig. S5. (**h**) Histogram shows the quantitative expression changes of Nrf2, NQO-1, and Prdx-1; all protein expression was normalised to a-tubulin. (**i,j**) Representative images and quantitative of immunofluorescence staining for Nrf2, NQO-1, and Prdx-1 (scale bar = 20 μm). Data are presented as the mean ± SEM (*n* = 3 in each group). *p < 0.05 vs. the control group; **p < 0.05 vs. the model group.

It is well known that excess oxidative stress is key pathophysiological process in cell death. The HE staining showed that DCM exhibited focal necrosis and infiltration of chronic inflammatory cells (black arrow), CVB-D significantly attenuated pathological changes (Fig. 2a). Mitochondria, as the main origin and attacking target of ROS, play a key role in energy metabolism. TEM revealed significant swelling and deformed mitochondria in DCM (green arrow), and CVB-D ameliorated the morphological abnormalities in the mitochondria (Fig. 2b).The mitoQ, a mitochondria-targeted antioxidant agent, could inhibit the mitochondria pathological damage in the DCM. 8-OHdG (red arrow, Fig. 2c,d), the biomarker of DNA oxidative damage, was significantly increase in DCM and attenuated by CVB-D. The SOD activity was significant decrease, on the contrary, increase MDA contents in DCM. Treated with CVB-D could alleviate (Fig. 2e,f).

Nrf2 plays a vital role in antioxidative responses through the up-regulation of multiple antioxidant components, such as NQO-1 and Prdx1. Western blotting results indicated that the protein expression of Nrf2, NQO-1, and Prdx1 were significantly decreased in DCM, however, treated with CVB-D increased Nrf2, NQO-1, and Prdx1 protein expression (Fig. 2g,h). These results were consistent with the immunofluorescence staining results (Fig. 2I,j). Taken together, CVB-D could ameliorate diabetes-induced oxidative stress which maybe involve in activating the Nrf2-related antioxidant pathways in DCM.

### CVB-D attenuates HG-induced oxidative damage in PNRCMs

**Figure 3:**
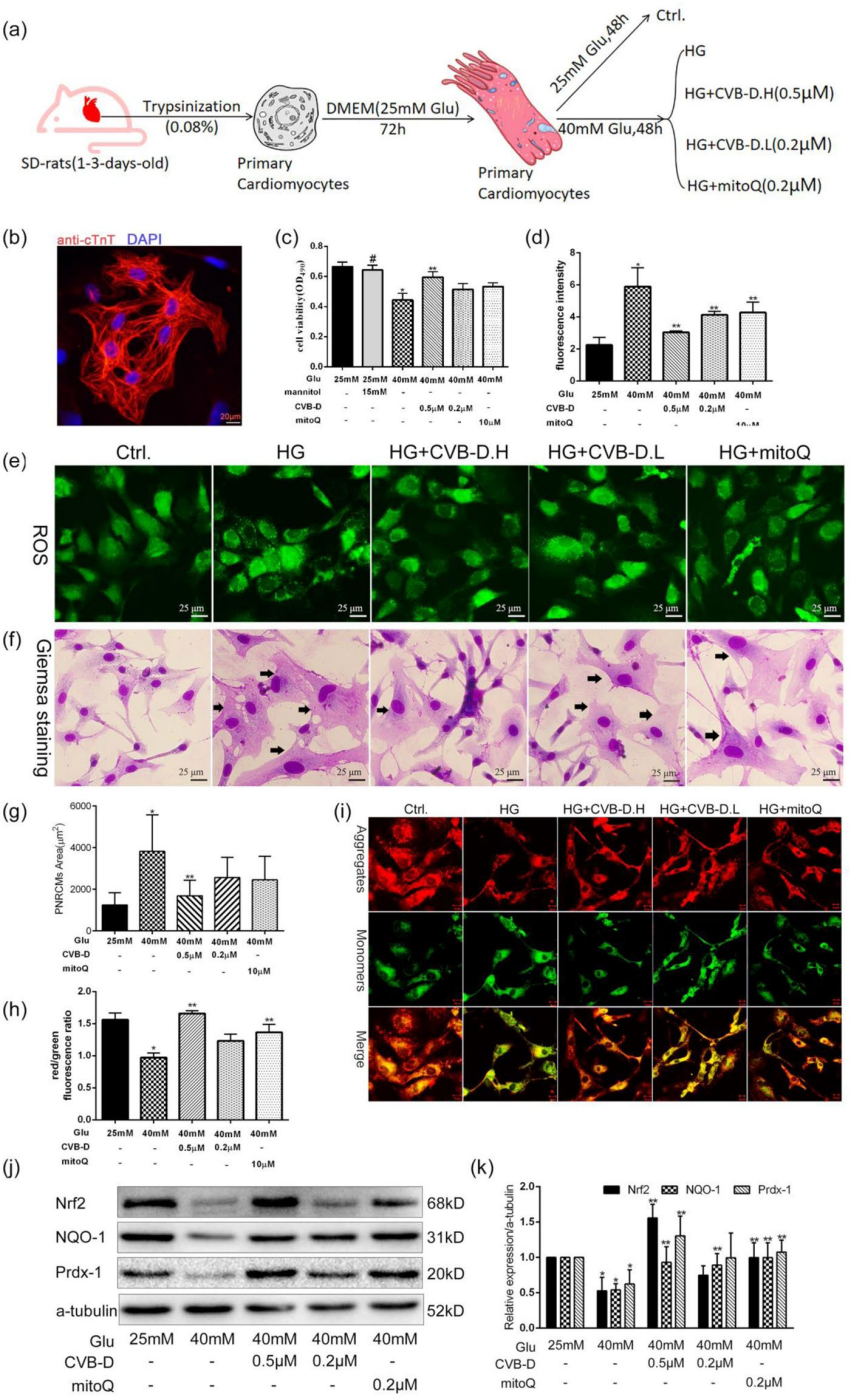
CVB-D ameliorates HG-induced oxidative damage in PNRCMs. (**a**)Experimental design. (**b**)Immunofluorescence images of PNRCMs stained for cTnT and the cell nuclei (with DAPI); scale bar = 25 μm. (**c**) MTT assay analysed the cell viability. (**d,e**) Analysis of ROS in PNRCMs by DCFH-DA probe (scale bar = 25 μm). (**f**) Giemsa staining was used to observe the morphological changes in PNRCMs (scale bar = 25 μm). (**g**) Quantitative analysis of PNRCMs size by using image J software.(**h,i**) The MMP, as analysed by JC-1 staining in PNRCMs (scale bar = 10 μm). Quantitative analysis of MMP in PNRCMs. (j,k) The protein levels of Nrf2, NQO-1, and Prdx-1 in PNRCMs assayed by western blotting analysis,full–length blots are presented in supplementary Fig. S6. Data are presented as the mean ± SEM (*n* = 3 in each group), *p < 0.05 vs. the control group; **p < 0.05 vs. the model group; ^#^*p* > 0.05 vs. the control group.

To investigate the potential mechanism of CVB-D-mediated antioxidant stress *in vitro*, the PNRCMs were exposed to 40 mM glucose (high-glucose,HG) medium which induced oxidative stress damage. PNRCMs were isolated according to a previously published protocol^26^ and the cardiomyocytes were purified by using a differential attachment methods and further identified by cardiac troponin T(cTnT) immunostaining (Fig. 3b). MTT results suggest that 25 mM glucose DMEM is beneficial to the survival of PNRCMs (Supplementary Fig. S2). Preincubated with CVB-D (0.5 µM) could ameliorated the cardiomyocytes viability induced by HG (Fig. 3c, Supplementary Fig. S3). The osmotic pressure control, 40 mM mannitol, did not affect the cell viability. CVB-D (0.2 and 0.5 µM) inhibited ROS generation (Fig. 3d,e) and partially reversed the decreasing mitochondrial membrane potential (Fig. 3h,i). CVB-D (0.5 µM) inhibited the cardiomyocytes hypertrophy (black arrow) induced by HG by Giemsa staining (Fig. 3f,g). Exposure the PNRCMs to high glucose, the protein expression of Nrf2, and downstream protein NQO-1 and Prdx1 were significantly down-regulation by western blotting. Preincubation with CVB-D, the protein level was restored in certain extent, suggesting that CVB-D activated Nrf2-related antioxidant signalling (Fig. 3j,k). Above all, CVB-D could ameliorate HG-induced oxidative stress via the activation of the Nrf2-related antioxidant pathway in PNRCMs.

### CVB-D attenuates HG-induced oxidative stress in PNRCMs via Nrf2 regulation

**Figure 4:**
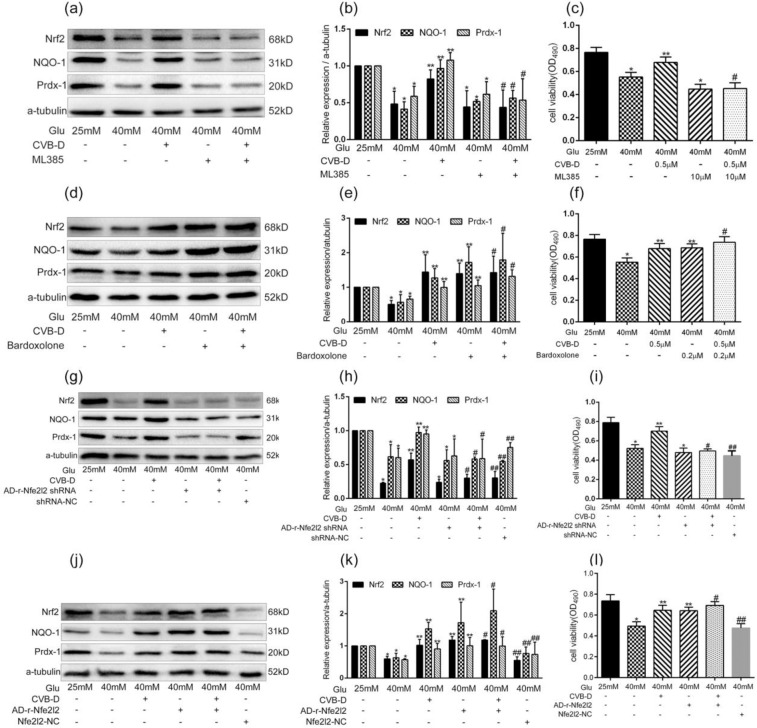
CVB-D ameliorates HG-induced oxidative damage in PNRCMs through Nrf2 regulation. (**a,b**) Western blotting analysis of protein levels of Nrf2, NQO-1, and Prdx-1 in PNRCMs after treatment with an Nrf2 inhibitor (ML385, 10 µM), full–length blots are presented in Supplementary Fig. S7. (**c**) MTT assay was used to analyse the cell viability after treatment with ML385. *p < 0.05 vs. the control group; **p < 0.05 vs. the model group; ^#^*p* > 0.05 vs. the HG + ML385 group. (**d,e**) Western blotting analysis of protein levels of Nrf2, NQO-1, and Prdx-1 in PNRCMs treated with Nrf2 activator (bardoxolone, 0.2 µM), full–length blots are presented in Supplementary Fig. S8. (f) The MTT assay was used to analyse the cell viability after treatment with bardoxolone. *p < 0.05 vs. the control group; **p < 0.05 vs. the model group; ^#^*p* > 0.05 vs. the bardoxolone group. (**g,h**) Western blotting analysis of protein levels of Nrf2, NQO-1, and Prdx-1 in PNRCMs after infection with Nrf2 shRNA adenovirus, full–length blots are presented in Supplementary Fig. S9. (**i**) The MTT assay was used to analyse the cell viability after infection with Nrf2 shRNA adenovirus. *p < 0.05 vs. the control group; **p < 0.05 vs. the model group; ^#^*p* > 0.05 vs. the HG + shRNA group; ^##^*p* > 0.05 vs. the model group. (**j,k**) Western blotting analysis of protein levels of Nrf2, NQO-1, and Prdx-1 in PNRCMs after infection with Nrf2 overexpression plasmid adenoviruses, full–length blots are presented in Supplementary Fig. S10. (**l**) The MTT assay was used to analyse the cell viability after infection with the Nrf2 overexpression plasmid adenoviruses. *p < 0.05 vs. control group; **p < 0.05 vs. model group; ^#^*p* > 0.05 vs. HG + Nrf2 overexpression group; ^##^*p* > 0.05 vs. model group. The data are presented as the mean ± SEM (*n* = 3 in each group).

To investigate the direct role of Nrf2 in CVB-D-mediated cardiac protection against HG, PNRCMs were pre-incubated with a pharmacological Nrf2 inhibitor (ML385) or activator (Bardoxolone) in with or without CVB-D^27,28^. The cell viability and the protein expression of Nrf2, NQO-1, and Prdx1 were determined. The protection effect of CVB-D was abrogated in the presence of ML385, which also clearly inhibited CVB-D-induced upregulation protein expression of Nrf2, NQO1, and Prdx1 (Fig. 4a–c). In addition, neither the cell viability nor the protein expression of Nrf2, NQO-1, and Prdx1 showed significant differences between the HG + bardoxolone and HG + Bardoxolone + CVB-D groups (Fig. 4d–f), suggesting that CVB-D protected against PNRCMs via the activation of Nrf2.

To verify the direct role of Nrf2 in mediating CVB-D at the genetic level, Nrf2 shRNA and overexpression plasmid adenoviruses were transfected into the PNRCMs. The results indicated that silencing of the Nrf2 gene abolished CVB-D-induced activation of Nrf2 and its downstream target proteins (Fig. 4g–i). The overexpression of Nrf2 increased the expression of Nrf2, NQO1, and Prdx1, as did CVB-D treatment. Moreover, the combined Nrf2 plasmid with CVB-D exerted a synergetic effect on increasing expression of NQO1 and Prdx1 (Fig. 4j–l). Our results demonstrated that the antioxidant property of CVB-D was mediated, at least partially, via activation of the Nrf2 pathway.

### CVB-D promotes Nrf2 nuclear translocation

**Figure 5:**
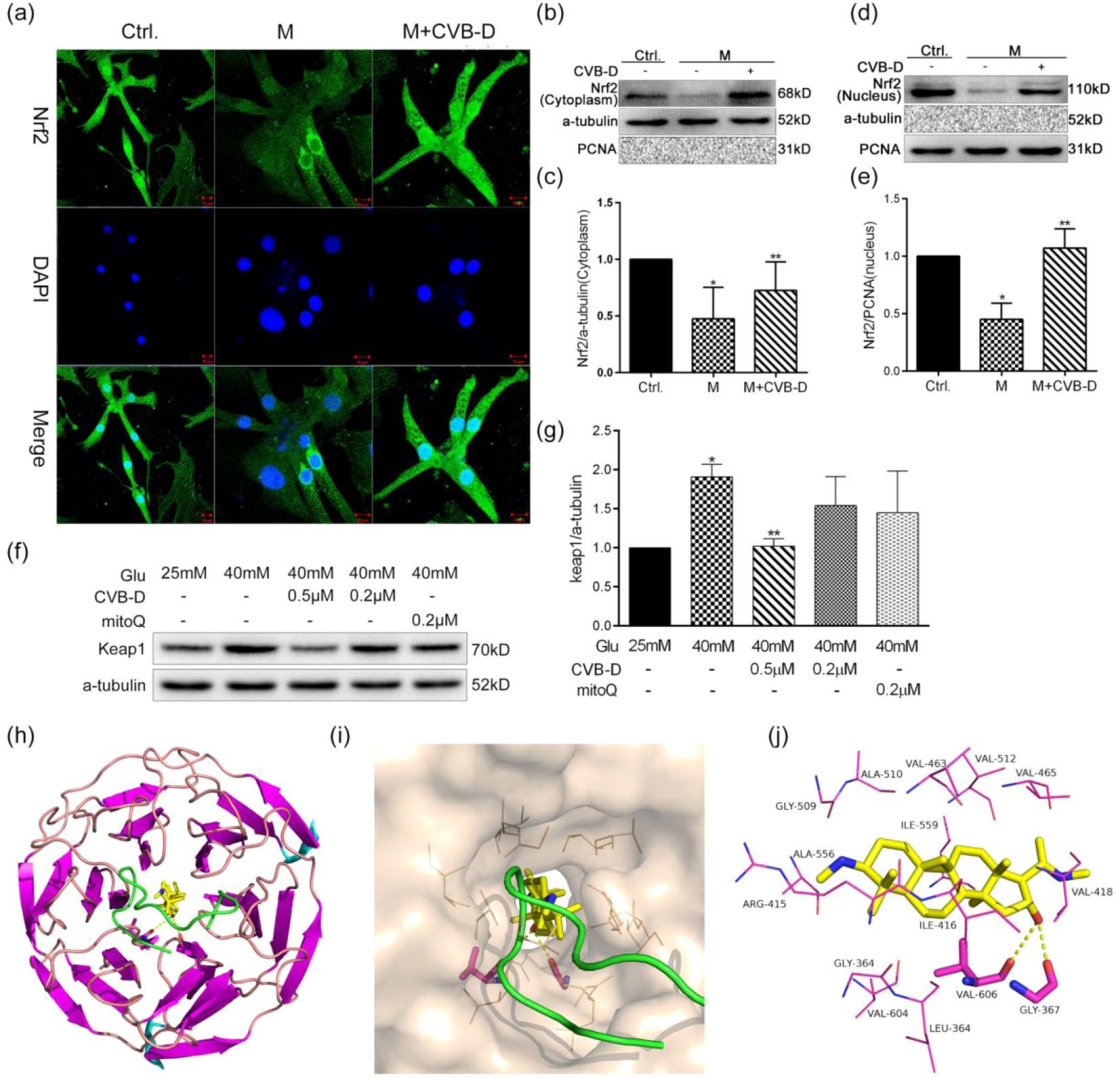
CVB-D promotes Nrf2 nuclear translocation. (**a**) Immunofluorescence image of PNRCMs stained for Nrf2 and the nuclear stain DAPI (scale bar = 10 μm). (**b–e**) The expression of cytoplasmic Nrf2 and nuclear Nrf2 was detected by western blotting. (**f,g**) The expression of Keap1 in PNRCMs was assayed by western blotting analysis, full–length blots of fig.b,d,f are presented in Supplementary Fig. S11. The data are presented as the mean ± SEM (*n* = 3 in each group), *p < 0.05 vs. the control group; **p < 0.05 vs. the model group. (**h–j**) Proposed docking modes of CVB-D in the binding site of Nrf2-Keap1 complex (PDB ID: 2flu).For h, panorama of protein binding with CVB-D. For i, CVB-D binding site of the Nrf2-Keap1 complex. For j, interactions of key residues of Nrf2-Keap1 complex with CVB-D.

The expression and activity of Nrf2 are regulated by its cullin3-based ubiquitin E3 ligase protein Keap1. Upon exposure to various stress conditions, Nrf2 is uncoupled from Keap1 and subsequently translocated to the nucleus, where it activates its downstream signals. To end this, we explore that CVB-D can promote the nuclear translocation of Nrf2 with immunofluorescence assay for Nrf2 and western blotting analysis of cytosolic and nuclear extracts were performed. The immunofluorescence assay showed that Nrf2 proteins predominantly accumulated around the nucleus after exposure to HG and treatment with CVB-D was closely related with the promotion on the nuclear translocation of Nrf2 (Fig. 5a). The results were further verified by the western blotting (Fig. 5b–e).

As the ubiquitinated ligase of Nrf2 protein, the expression of keap1 is closely related to the nuclear translocation of Nrf2. As shown in Fig. 5f,g, unlike in the control group, the protein expression of Keap1 was increased after exposure to 40 mM glucose and was attenuated by the addition of CVB-D, suggesting that the nuclear translocation of Nrf2 was at least partly dependent on the Keap1 protein expression. Interestingly, the *in vivo* data is consistent with the results of *in vitro* experiments (Supplementary Fig. S4).

To further clarify the interaction between CVB-D and the Nrf2-Keap1 complex, we performed a docking study using AutoDock Vina 1.1.2, and calculated their binding free energy by using the MM/GBSA method. In Fig. 5h–j, CVB-D was located in the Nrf2-Keap1 binding pocket, in which CVB-D forms one double hydrogens bonding with residue GLY-367 and VAL-606.MMGBSA showed that the binding free energy between Nrf2 and keap1 was −66.4 kcal/mol, and the main contribution was from electrostatic and vdW interactions. In addition, polar solvation was unfavourable for this binding. However, when CVB-D was present, the binding free energy between Nrf2 and Keap1 was changed to −56.7 kcal/mol, almost lower 10 cal/mol than in the absence of CVB-D (Supplementary Material, Table 1). This observation suggested that CVB-D could suppress the binding between Nrf2 and Keap1, which is an important factor for Nrf2 nuclear translocation. These results demonstrated the potential action of CVB-D as an enhancer of Nrf2 nuclear translocation, dependent on the down-regulation of the protein expression of keap1, and the reduced binding free energy of the Nrf2-Keap1 complex.

### CVB-D attenuates H_2_O_2_-induced oxidative stress damage in PNRCMs

**Figure 6:**
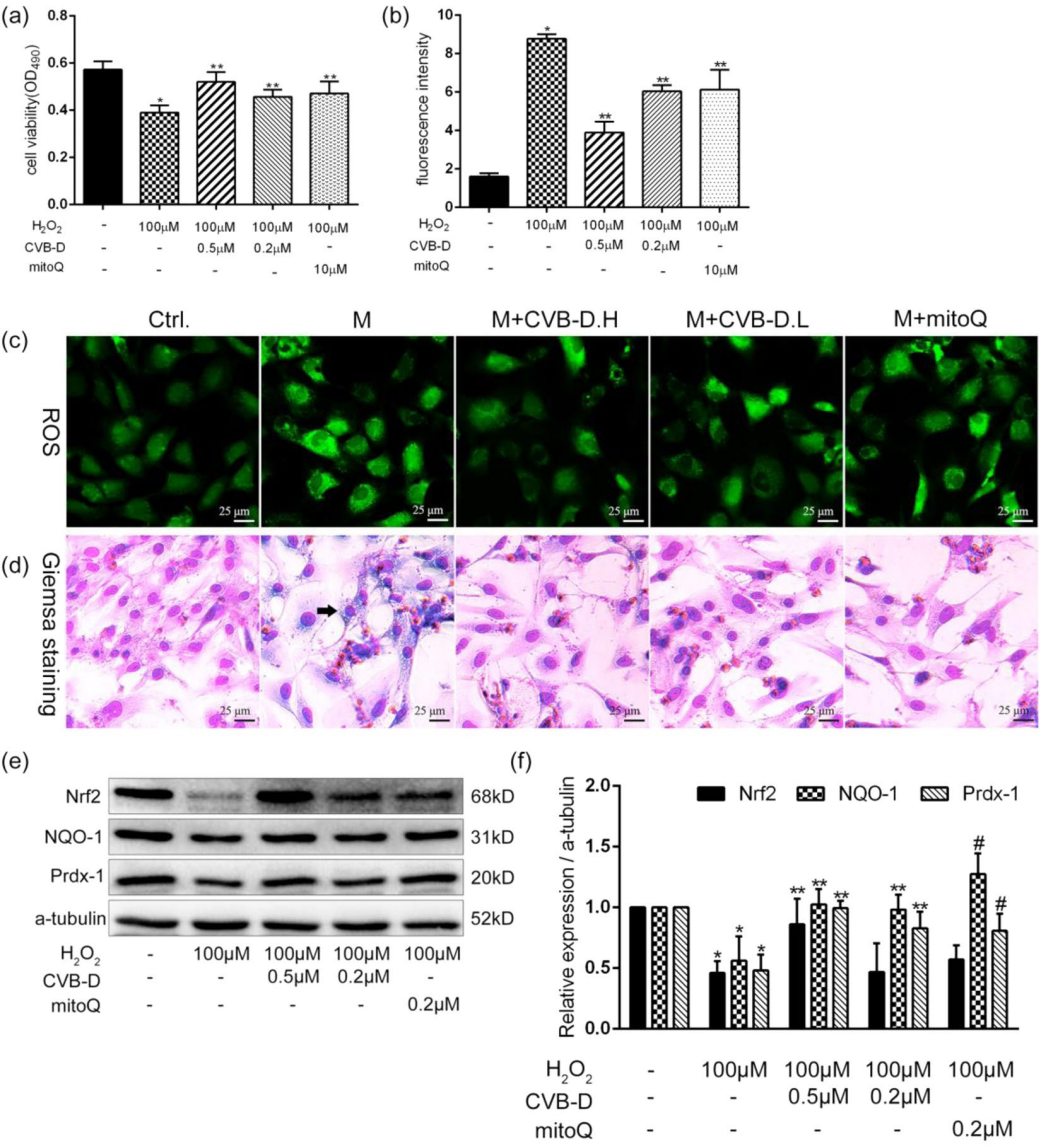
CVB-D attenuates H_2_O_2_-induced oxidative damage in PNRCMs. (**a**) The MTT assay was used to analyse cell viability. (**b,c**) The detection of ROS by the DCFH-DA probe (scale bar = 25 μm). (**d**) Giemsa staining was used to observe the morphological changes of PNRCMs (scale bar = 25 μm). (**e,f**) Protein expression of Nrf2, NQO-1, and Prdx-1 was assayed by western blotting, full–length blots are presented in Supplementary Fig. S12. The data are presented as the mean ± SEM (*n* = 3 in each group),*p < 0.05 versus the control group; **p < 0.05 versus the model group.

To further explore the mechanism of CVB-D action, PNRCMs was exposed to H_2_O_2_, a common method to analyse oxidative stress^29,30^. The MTT assay results indicated that CVB-D significantly attenuated H_2_O_2_ (100 µM, 24 h) induced toxicity to cardiomyocytes (Fig. 6a), and that CVB-D also inhibited ROS generation (Fig. 6b,c) similar to the HG model. Unlike cells exposed to HG, hydrogen peroxide causes vacuolar degeneration of PNRCMs (Fig. 6d, black arrow). It was found that the trends in Nrf2, NQO-1, and Prdx1 were consistent with the trends of HG model (Fig. 6e,f). These results were further confirming the antioxidant effect of CVB-D.

## Discussion

DCM is a special form of heart disease that was first described in 1972 and remains the principal cause of death in patients with diabetes^31^. As the most common complication of diabetes, despite the application of conventional treatment strategies, such as hypoglycaemic, lipid-lowering, and anti-hypertensive agents, the incidence of DCM in patients with diabetic is still higher^4,32^. Therefore, a novel effective therapeutic agent is urgently required. CVB-D has been demonstrated multiple pharmacological activities and widely applied in cardiovascular diseases^22–25^. However, the possible beneficial effects and potential mechanism of CVB-D in T_2_DM-associated cardiomyopathy remains unknown. Our results has provided the first demonstration that CVB-D could effectively ameliorates cardiac dysfunction and increases survival in rats with DCM, and was associated with the activation of Nrf2 and its associated signalling targets.

DCM is usually characterized by cardiac structural and functional disorders. Some risk factors, such as oxidative stress and inflammation, promote cardiomyocyte death, cardiac tissue interstitial fibrosis, and cardiac stiffness, leading to diastolic and systolic dysfunction, and, eventually heart failure^2,33,34^. However, the exact pathological process of DCM is still unclear and there are no clear diagnostic indicators^35^. At present study, we evaluated the EF of rats by non-invasive electrocardiograph and, simultaneously, plasma BNP levels to confirm the occurrence of heart failure. After 12 weeks of elevated blood glucose, the ejection fraction (EF = 65.4% ± 1.8%) of diabetic and the BNP level (153.2 ± 15 pg/mL) are significantly decreased and increased compared to control group, indicating that the heart failure model was successfully established^36^. Meanwhile, the results of TEM revealed significant mitochondrial disruption in DCM; MDA and a biomarker of DNA damage (8-OHdG) were significantly increased, and SOD activity was decreased in DCM hearts. All findings were consistent with those of previous studies^37,38^ suggesting that oxidative stress is one of the key important pathophysiological factor in DCM. The CVB-D can ameliorate oxidative stress damage in DCM, which suggests potential beneficial effect of CVB-D on DCM via ameliorating oxidative stress effect.

As a major transcription factor of cellular antioxidant response, accumulated evidence has suggested that activation of Nrf2 may have potential therapeutic in multiple cardiovascular diseases, such as heart failure, diabetes, diabetic nephropathy, and DCM^39–42^. At present, diabetes caused a significant down-regulation of Nrf2 and downstream molecules such as NQO-1 and Prdx-1, and CVB-D alleviated the expression of these proteins both *in vivo* and *in vitro*. To further explore whether the protective efficacy of CVB-D was related to Nrf2, the inhibitor/activator or sh-RNA/overexpression plasmids for Nrf2 were highlighted on HG challenged to PNRCMs and the results indicated that CVB-D protected PNRCMs via the activation of Nrf2. As well known, Nrf2 activators promote the expression of Nrf2 by disrupting the interaction between Keap1 and Nrf2^43,44^. Our results confirmed that CVB-D increased Nrf2 expression in the nucleus and reduced the protein expression of Keap1 both *in vivo* and *in vitro*, and molecular docking revealed that the binding free energy between Nrf2 and Keap1 was reduced by approximately 15% in the presence of CVB-D. Therefore, we concluded the CVB-D-mediated increase in Nrf2 expression via the inhibition of Keap1 expression and the decrease in binding free energy of the Nrf2- Keap1 complex, which represented a novel method that CVB-D promotes the uncoupling mechanism of Nrf2 and Keap1.

Hyperglycaemia is one of the causes of oxidative stress. To further clarify the antioxidant capacity of CVB-D, a classical oxidative stress model must be tested. It is well known that H_2_O_2_ is a potent inducer of oxidative stress and an ideal model for studying the antioxidant effects of CVB-D^45,46^. Unlike cardiac hypertrophy induced by hyperglycaemia, H_2_O_2_ leads to vacuolar degeneration of cardiomyocytes, and produces higher levels of ROS. Surprisingly, CVB-D demonstrated favourable anti-oxidant stress effect in both HG and H_2_O_2_ models.

**Figure 7:**
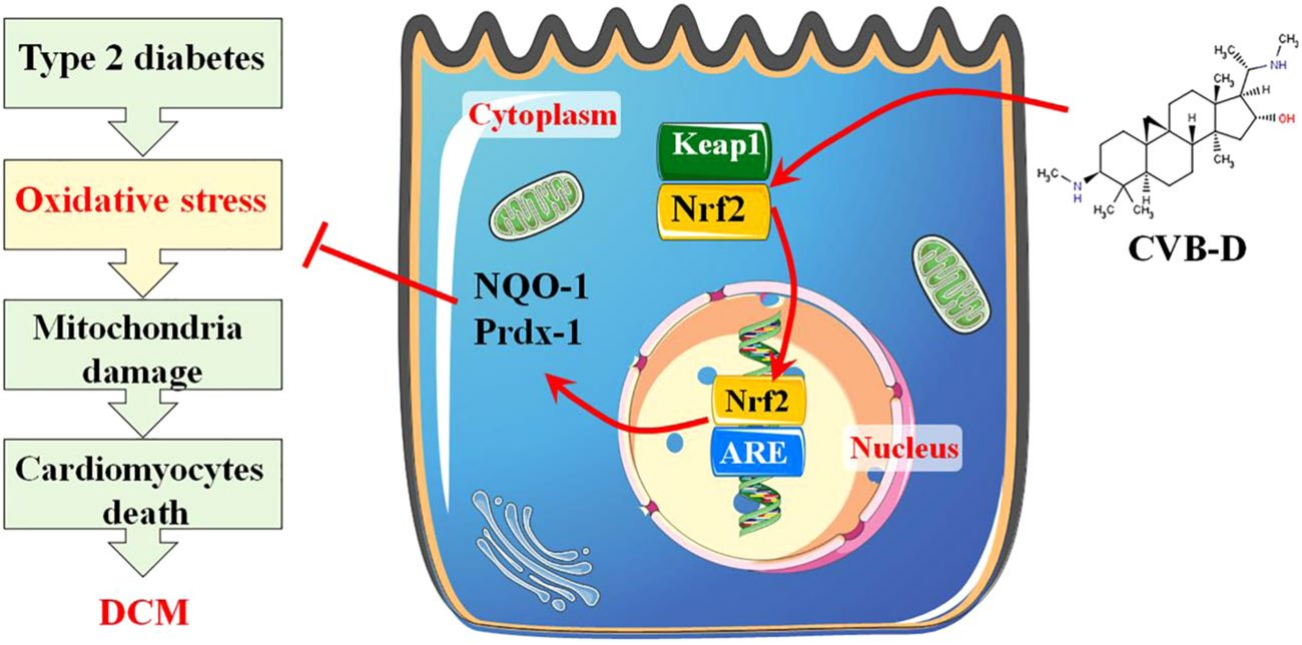
Scheme summarising the protective effect of CVB-D against DCM and the underlying mechanisms. CVB-D treatment significantly improved cardiac function via the amelioration of oxidative damage in DCM, which was associated with the promotion of Nrf2 nuclear translocation and the activation of Nrf2 downstream antioxidative stress signalling targets (NQO-1 and Prdx1).

In summary, the present data is the first time that CVB-D could ameliorate the oxidative stress in DCM both *in vivo* and *in vitro*. CVB-D can inhibit the expression of keap1 and reduce the free binding energy of the Nrf2-Keap1 complex, promote Nrf2 translocating into the nucleus, activate its downstream signalling targets (NQO-1, and prdx1), and exert attenuating oxidative stress effects (Fig. 7). Our findings also have positive implications on the novel promising clinical applications of CVB-D.

## Methods

### Chemicals and reagents

CVB-D (purity >98%; the chemical structure is shown in Fig. 1a, catalog number: PS1540–0050) was obtained from PUSH BIO-TECHNOLOGY Co. Ltd (Chengdu, China). CVB-D tablet (0.5 mg/tablet, catalog number: 18020601) was purchased from MingRen pharmacy Co. Ltd (Kaifeng, China). Mitoquinone mesylate (reference drug, Cat. NO.: HY-100116A)^47^, ML385 (Nrf2 inhibitor, catalogue number: HY-100523) and bardoxolone (Nrf2 activator, catalogue number: HY-14909) were obtained from MCE China (Shanghai, China). High fat high sucrose diet (HFS):nutrient components: 18% fat, 20% sucrose, 2% cholesterol, 0.2% cholate, 59.8% normal diet; calorie percentage:44.5% fat,16.5% sucrose,8% protein,30.9% carbohydrate (Tengxin Biotechnology Inc., Chongqing, China). Streptozocin (STZ, catalogue number: 18883-66-4) was purchased from Sigma (St Louis, MO, USA). Primary antibodies against Nrf2 (16396-1-AP), Keap1 (10503-2-AP), Prdx1 (15816-1-AP), and PCNA (10205-2-AP) were purchased from Proteintech (Chicago, USA). Primary antibodies against NQO-1(ab34173), a-tubulin(ab52866), 8-hydroxy-2′-deoxyguanosine (8-OHdG, ab48508) and rat plasma brain natriuretic peptide (BNP, ab108816) ELISA kit were obtained from Abcam (Cambridge, USA). The HRP-conjugated secondary antibodies(A0192),RIPA buffer(P0013B) were obtained from Beyotime (Jiangsu, China). ELISA kits for the measurement of fasting insulin, malondialdehyde (MDA, E-EL-0060c), and superoxide dismutase (SOD, E-BC-K022-M) levels were obtained from Elabscience Co. Ltd. (Shanghai, China). The ROS Assay Kit (DCFH-DA fluorescent probe, D6470), Nuclear and Cytoplasm Protein Extraction Kit (R0050), and Mitochondrial Membrane Potential (MMP, M8650) Assay Kit with JC-1 were obtained from Solarbio (Beijing, China).

### DCM rat model reproduction and treatment

Animal experiments were approved by the Ethic Committee of Guizhou Medical University (Guizhou, China). All animal protocols were performed in accordance with the *NIH Guide for the Care and Use of Laboratory Animals* (NIH Publication No. 85-23, revised 1996). A total of 120, health female Sprague-Dawley (SD) rats (8-week-old, body weight 180–200 g) were obtained from the experimental animal center of Guizhou Medical University. The rats were divided into two groups as following: the control group (n = 24, normal diet) and the HFS group (n = 96), HFS group was fed daily with HFS to induce insulin resistance as previously described^48^, Control group fed with a standard chow diet containing 16% protein, 4% fat and 60% carbohydrate. All rats were maintained and fed ad libitum under a controlled 12/12 hour light/dark cycle and temperature (22 ± 2 °C) for 24 weeks. After 12 weeks of feeding, the serum samples of rats from each group were taken from the tail vein. Fasting blood glucose (FBG) and fasting insulin (INS) levels were analysed to evaluate insulin resistance. Insulin resistance (HOMA-IR) was calculated as follows: HOMA-IR =  (FBG*INS)/22.5. Then, Model rats that have developed insulin resistance were treated with streptozotocin by a single intraperitoneal injection of 30 mg/kg in 10 mg/ml sodium citrate buffer (pH 4.5), Control group was injected with the same volume of sodium citrate buffer.After STZ injection for 3 days, diabetic model was considered successful as the FBG level was more than 11.1 mM. Then, the diabetic rats were randomly divided into four groups as following: DCM; DCM + CVB-D.H (2 mg/kg/day, ig); DCM + CVB-D.L (1 mg/kg/day, ig); DCM + mitoQ (reference drug, 5 mg/kg twice weekly, ip), the control group and DCM group were administered saline for the next 12 weeks (Fig. 1b), and plasma was taken from the rat tail vein to measure the FBG and BNP levels every month. The creatine kinase isoenzyme MB (CK-MB) and Lactic dehydrogenase 1 (LDH1) were detected in serum by commercial kits in accordance with the manual instruction at the end of experiment.

#### Cardiac function evaluation

Cardiac ultrasonography and serum BNP levels were assessed monthly to confirm the successful establishment of DCM^49^. The rats were anaesthetised with inhalation of sevoflurane. The left ventricular ejection fraction (EF), left ventricular fractional shortening (LVFS), left ventricular mass(LVmass), left ventricular posterior wall thickness diastole (LVPWd) and left ventricular posterior wall thickness systole (LVPWs) were determined and analysed M-mode echocardiography with a 30 MHz linear transducer using a VINNO echocardiography system (SUZHOU, CHINA) which was evaluated the cardiac function. The DCM model was successfully reproduced by the low EF and high BNP levels compared to control group.

#### Morphological analysis of cardiac tissue

The cardiac tissue samples were fixed in 4% buffered paraformaldehyde, cut to a thickness of 5 μm, stained with H&E, and imaged by using a Nikon microscope (Nikon, Japan). To observe the ultrastructure of mitochondria in the cardiomyocytes, the left ventricular sample was cut into 1 mm blocks, fixed with 4% paraformaldehyde solution containing 2.5% glutaraldehyde, and then imaged by using an electron microscope (JEM-1001, JEOL Ltd., Tokyo, Japan).

#### Measurement of SOD activity and MDA content

Total proteins were extracted from cardiac tissues lysed in lysis buffer. Protein concentration was meansured by Bicinchoninic acid method. The SOD activity and MDA contents were measured by commercial kits in accordance with the manufacturer’s method.

#### IHC/IF

The 8-OHdG, Nrf2, NQO-1, Prdx1 were evaluated by immunohistochemical (IHC) or Immunofluorescence (IF) staining of heart tissue. The positive areas were detected by using ImageJ (U.S. National Institutes of Health, Bethesda, MD).

### Cell culture

#### The primary neonatal rat cardiomyocyte culture

The primary neonatal rat cardiomyocytes (PNRCMs) were separated and purified from the ventricles of new-born 1–2-day-old SD rats, as previously described^26^. PNRCMs were cultured in 25 mM glucose DMEM (containing 10% FBS) in an incubator (5% CO_2_, 37 °C) for 72 h. The cells were identified as cardiomyocytes by immunofluorescence staining of cardiac troponin (cTnT).

#### Cell treatment

To investigate the effects of CVB-D in high glucose (HG)-induced injury of PNRCMs, cardiomyocytes were randomly divided into five groups as following: (1) Control (25 mM glucose); (2) Model (40 mM glucose); (3) M + CVB-D.H (0.5 µM); (4) M + CVB-D.L (0.2 µM); and (5) M + mitoQ (0.2 µM). PNRCMs were pre-treated with CVB-D or mitoQ for 2 h, and then cultured with 40 mM glucose for an additional 48 h (Fig. 3a). Equal concentration of mannitol were evaluated the effect of osmotic pressure on cell viability.

To investigate the ameliorating oxidative stress effect of CVB-D, H_2_O_2_-induced cytotoxicity in PNRCMs was established, and the groups were design as following: (1) Control; (2) M (100 µM H_2_O_2_); (3) M + CVB-D.H (0.5 µM); (4) M + CVB-D.L (0.2 µM); and (5) M + mitoQ (0.2 µM). The PNRCMs were pre-treated with CVB-D or mitoQ for 2 h, followed exposure to 100 µM H_2_O_2_ 24 h.

The role of Nrf2 was investigated in the protection of CVB-D on cardiomyocytes challenged to HG, The cardiomyocytes were incubated with or without agonists/inhibitors of Nrf2. Adenoviruses expressing short hairpin (sh) RNA or Nrf2-overexpressing plasmids were further explore the molecular mechanism of CVB-D.

#### Adenovirus transfection

Adenoviruses expressing a short hairpin (sh) RNA (AD-r-Nfe2l2 shRNA-EGFP) or overexpression plasmid (NM___031789, named as AD-r- Nfe2l2–3xflag-EGFP) targeted to Nrf2, and the control vectors, were obtained from Hanbio Technology Ltd (Shanghai, China). The shRNA sequence targeting for Nrf2 was: TcgagGGGTAAGTCGAGAAGTGTTTGTGCTTAACACTTCTCGACTTACCCTTTTTTa. The MOI of adenoviruses was 30:1 in the present study.

#### The measurement of cell viability

Cell viability was analysed using MTT assay. The PNRCMs were seeded into 96-well plates at 5000 cells/well and cultured for 72 h. At the designing experimental time point, the MTT solution (5 mg/mL, 20 µL/well) was added to the well and then incubated for 4 h. The absorbance at 490 nm was measured with Varioskan Flash (Thermo fisher Scientific, Finland).

#### Giemsa staining

After indicated incubation, Giemsa staining and cell morphology was examined under an inversion microscope (Leica DMi8, Germany). The cell area of each group was measured by the software of the Image J Software, each group randomly selected 50 cells and calculated the average value.

#### Measurement of ROS

The production of ROS was measured by detecting the fluorescent intensity of oxidant-sensitive probe DCFH-DA, which was a stable nonfluorescent molecule that passively diffuses into cells, where the acetate could be cleaved by intracellular esterase to produce a polar diol that was well retained within in the cells. After the cell treatment with the designing protocol, which was loaded with DCFH-DA (10 μM) as per the manufacturer’s instruction for 30 min without light. The fluorescent intensity was recorded by excitation at 488 nm and emission at 525 nm using a Varioskan Flash (Thermo fisher Scientific, Finland). Images were observed under a fluorescence microscope (Leica DMi8, Germany).

#### Determination of MMP by JC-1 fluorescent staining

PNRCMs were exposed to 5,5′,6,6′-tetrachloro-1,1′,3,3′-tetraethyl benzimidazolyl-carbocyanine iodide (JC-1) dye (10 μM) for 40 min at 37 °C after each treatment. The detection wavelengths for the JC-1 aggregate were 543 (excitation) and 590 (emission) nm and the detection wavelengths of the JC-1 monomer were 488 (excitation) and 525 nm (Thermo fisher Scientific, Finland). The depolarisation of the mitochondrial membrane was determined from the ratio of red fluorescence to green fluorescence. Images were viewed and captured by using a fluorescence microscope (Leica DMi8, Germany).

#### Immunofluorescence analysis

Immunofluorescence staining of PNRCMs with anti-Nrf2 antibody (1:100 dilutions) was performed to observe the nuclear translocations of Nrf2. Images of cells were captured by using a confocal microscope (ZEISS, LSM710).

### Western blotting analysis

Protein samples were extracted from cardiac tissues and PNRCMs. About 50 mg of heart tissue was mixed with 300 µL RIPA buffer [50 mM Tris(pH 7.4),150 mM NaCl,1% Triton X-100,1% sodium deoxycholate,0.1% SDS,sodium orthovanadate,sodium fluoride,EDTA,leupeptin] and manually ground on ice using a glass homogenizer. The homogenate was centrifuged(4 °C,12000 g,15 min) and the supernatant was taken for subsequent experiments. Protein quantification was detected by BCA commercial kit, 30 µg proteins were separated by using 10% SDS-PAGE and transferred to PVDF membranes. After non-specific binding to the membrane was blocked with 5% BSA, the membranes were incubated with the following primary antibodies: Nrf2, Keap1, NQO-1, Prdx-1, and a-tubulin, PCNA (dilutions of 1:1000, 1:1000, 1:1000, 1:1000, 1:10000, and 1:5000, respectively) overnight at 4 °C. Subsequently, after washing, the membranes were incubated with the appropriate secondary antibody (dilution 1:10000) for 2 h at room temperature (about 20 °C ± 5). The immunoreactive bands were scanned and quantified by using ChemiDoc^TM^ MP System (Bio-Rad).

### Molecular docking analysis

The 3D-structure of the Nrf2-Keap1 complex (PDB ID: 2flu) was download from the PDB database. AutoDockTool 1.1.2 was used to determine the atom types and calculate the atomic charges of the receptor protein, and a.pdbqt file was saved for the docking analysis. The 2D structure of the ligand (CVB-D) was drawn by ChemDraw, and saved as a.cdx file. Then, the MM2 force field in Chem3D was used to optimise the 3D structure of the ligand. AutoDockTool was used to determine the atom types and calculate the partial charges of the ligand, and a.pdbqt file was saved for the docking analysis. Docking was simulated by using AutoDock Vina 1.1.2.

The Molecular Mechanics Generalized Born Surface Area (MM/GBSA) module was used to analyse the change in binding energy of the Nrf2-Keap1 complex before and after CVB-D insertion. Pre-treatment of Nrf2-Keap1 crystals involved the removal of crystal water, addition of protein force fields, addition of antagonist ions, addition of solvation cartridges, and preferences. Subsequently, a 50 ns molecular dynamics simulation was performed on the complex and the binding free energy of the Nrf2-keap1 complex with the CVB-D-Nrf2-Keap1-ligand ternary complex was calculated.

### Statistical analysis

Data from a minimum of at least three independent experiments were calculated and expressed as the mean ± SEM. Statistical analysis of the data was evaluated using one-way ANOVA with Tukey *post hoc* test by using Prism 6.0 (GraphPad Software, San Diego,CA), Kaplan-Meier survival curves and Mantel-Cox log rank test were used to analysis the survival rates for rats, A *p* value < 0.05 was considered statistically significant.

## Data Availability

The datasets used and/or analyzed during the current study are available from the corresponding author on reasonable request.

## Supporting information

Supporting information.

## References

[1] Lundbaek K (1954). Diabetic angiopathy: a specific vascular disease. Lancet..

[2] Rubler S (1972). New type of cardiomyopathy associated with diabetic glomerulosclerosis. Am. J. Cardiol..

[3] Kannel WB, Hjortland M, Castelli WP (1974). Role of diabetes in congestive heart failure: the Framingham study. Am. J. Cardiol..

[4] Low Wang CC, Hess CN, Hiatt WR, Goldfine AB (2016). Clinical Update: Cardiovascular Disease in Diabetes Mellitus: Atherosclerotic Cardiovascular Disease and Heart Failure in Type 2 Diabetes Mellitus - Mechanisms, Management, and Clinical Considerations. Circulation.

[5] Zhang H, Chen X, Zong B (2018). Gypenosides improve diabetic cardiomyopathy by inhibiting ROS-mediated NLRP3 inflammasome activation. J. Cell Mol. Med..

[6] Huang X, Liu S, Wu D (2018). Facilitated Ca2+ homeostasis and attenuated myocardial autophagy contribute to alleviation of diabetic cardiomyopathy after bariatric surgery. Am. J. Physiol. Heart Circ. Physiol.

[7] Yang F, Li A, Qin Y (2019). A Novel Circular RNA Mediates Pyroptosis of Diabetic Cardiomyopathy by Functioning as a Competing Endogenous RNA. Mol. Ther. Nucleic Acids.

[8] Thandavarayan RA, Giridharan VV, Watanabe K, Konishi T (2011). Diabetic cardiomyopathy and oxidative stress: Role of antioxidants. Cardiovasc. Hematol. Agents Med. Chem..

[9] Wilson AJ (2018). Reactive oxygen species signaling in the diabetic heart: emerging prospect for therapeutic targeting. Heart.

[10] Jung KA, Kwak MK (2010). The Nrf2 system as a potential target for the development of indirect antioxidants. Molecules.

[11] Suzuki T, Yamamoto M (2017). Stress-sensing mechanisms and the physiological roles of the Keap1-Nrf2 system during cellular stress. J. Biol. Chem..

[12] Mills EL (2018). Itaconate is an anti-inflammatory metabolite that activates Nrf2 via alkylation of KEAP1. Nature.

[13] Goh KY, He L, Song J (2019). Mitoquinone ameliorates pressure overload-induced cardiac fibrosis and left ventricular dysfunction in mice. Redox Biol..

[14] Zhang T, Wu P, Budbazar E (2019). Mitophagy Reduces Oxidative Stress Via Keap1 (Kelch-Like Epichlorohydrin-Associated Protein 1)/Nrf2 (Nuclear Factor-E2-Related Factor 2)/PHB2 (Prohibitin 2) Pathway After Subarachnoid Hemorrhage in Rats. Stroke.

[15] Tian C, Gao L, Zhang A (2019). Therapeutic Effects of Nrf2 Activation by Bardoxolone Methyl in Chronic Heart Failure. J. Pharmacol. Exp. Ther..

[16] Sun Y, Zhou S, Guo H (2020). Protective effects of sulforaphane on type 2 diabetes-induced cardiomyopathy via AMPK-mediated activation of lipid metabolic pathways and NRF2 function. Metabolism.

[17] Chen QM, Maltagliati AJ (2018). Nrf2 at the heart of oxidative stress and cardiac protection. Physiol. Genomics.

[18] Ge ZD, Lian Q, Mao X, Xia Z (2019). Current Status and Challenges of NRF2 as a Potential Therapeutic Target for Diabetic Cardiomyopathy. Int. Heart J..

[19] Park BK (2011). Managing the challenge of chemically reactive metabolites in drug development. Nat. Rev. Drug. Discov..

[20] Chen QW, Shan HL, Sun HL, Wang H, Yang BF (2004). Effects of cyclovirobuxine D on intracellular Ca2+ and L-type Ca2+ current in rat ventricular cardiomyocytes. Acta Pharmaceutica Sin..

[21] Wang YX, Liu JW, Tau YH, Sheng BH (1989). Anti-arrhythmic action of cycloprotobuxine-A. Acta Pharmacologica Sin..

[22] Yu B, Fang TH, Lü GH, Xu HQ, Lu JF (2011). Beneficial effect of Cyclovirobuxine D on heart failure rats following myocardial infarction. Fitoterapia.

[23] Guo Q (2015). Cyclovirobuxine D Attenuates Doxorubicin-Induced Cardiomyopathy by Suppression of Oxidative Damage and Mitochondrial Biogenesis Impairment. Oxid. Med. Cell. Longev..

[24] Lu J (2014). Cyclovirobuxine D induces autophagy-associated cell death via the Akt/mTOR pathway in MCF-7 human breast cancer cells. J. Pharmacol. Sci..

[25] Wu JB (2017). Cyclovirobuxinum D alleviates cardiac hypertrophy in hyperthyroid rats by preventing apoptosis of cardiac cells and inhibiting the p38 mitogen-activated protein kinase signaling pathway. Chin. J. Integr. Med..

[26] Nguyen PD, Hsiao ST, Sivakumaran P, Lim SY, Dilley RJ (2012). Enrichment of neonatal rat cardiomyocytes in primary culture facilitates long-term maintenanceof contractility *in vitro*. Am. J. Physiol. Cell Physiol.

[27] Huang XT (2019). Dihydroartemisinin attenuates lipopolysaccharide induced acute lung injury in mice by suppressing NFκB signaling in an Nrf2 dependent manner. Int. J. Mol. Med..

[28] Song MK (2019). Bardoxolone ameliorates TGF-β1-associated renal fibrosis through Nrf2/Smad7 elevation. Free. Radic. Biol. Med..

[29] Khoshsirat S (2019). Protective effect of Photobiomodulation Therapy and Bone Marrow Stromal Stem Cells Conditioned Media on Pheochromocytoma Cell Line 12 Against Oxidative Stress Induced by Hydrogen Peroxide. J. Lasers Med. Sci..

[30] Zhang X, Lee MD, Wilson C, McCarron JG (2019). Hydrogen peroxide depolarizes mitochondria and inhibits IP3-evoked Ca2+ release in the endothelium of intact arteries. Cell Calcium.

[31] Hippisley-Cox J, Coupland C (2016). Diabetes treatments and risk of heart failure, cardiovascular disease, and all cause mortality: cohort study in primary care. BMJ.

[32] Rawshani A (2018). Risk Factors, Mortality, and Cardiovascular Outcomes in Patients with Type 2 Diabetes. N. Engl. J. Med..

[33] Levelt E (2016). Relationship Between Left Ventricular Structural and Metabolic Remodeling in Type 2 Diabetes. Diabetes.

[34] Palmieri V (2001). Effect of type 2 diabetes mellitus on left ventricular geometry and systolic function in hypertensive subjects: Hypertension Genetic Epidemiology Network (HyperGEN) study. Circulation.

[35] Lavanya A, Gaurav S G, Gerald P M, Eylem L (2019). Diabetic cardiomyopathy: Pathophysiology, theories and evidence to date. World J. Diabetes.

[36] Lam CS, Voors AA, de Boer RA, Solomon SD, van Veldhuisen DJ (2018). Heart Failure with Preserved Ejection Fraction: From Mechanisms To Therapies. Eur. Heart J..

[37] Zhao MX (2017). Salusin-β contributes to oxidative stress and inflammation in diabetic cardiomyopathy. Cell Death Dis..

[38] Tang SG (2019). Trimetazidine prevents diabetic cardiomyopathy by inhibiting Nox2/TRPC3-induced oxidative stress. J. Pharmacol. Sci..

[39] Chen QM, Maltagliati AJ (2018). Nrf2 at the heart of oxidative stress and cardiac protection. Physiol. Genomics.

[40] Wang F (2019). Sanbai Melon Seed Oil Exerts Its Protective Effects in a Diabetes Mellitus Model via the Akt/GSK-3β/Nrf2 Pathway. J. Diabetes Res..

[41] Wu H, Liu G, He Y, Da J, Xie B (2019). Obeticholic acid protects against diabetic cardiomyopathy by activation of FXR/Nrf2 signaling in db/db mice. Eur. J. Pharmacol..

[42] Landis RC, Quimby KR, Greenidge AR (2018). M1/M2 Macrophages in Diabetic Nephropathy: Nrf2/HO-1 as Therapeutic Targets. Curr. Pharm. Des..

[43] Zhuang CL, Wu ZL, Xing CG, Miao ZY (2017). Small molecules inhibiting Keap1-Nrf2 protein-protein interactions: a novel approach to activate Nrf2 function. Medchemcomm.

[44] Pallesen JS, Tran KT, Bach A (2018). Non-covalent small-molecule kelch-like ECH-associated protein 1-nuclear factor erythroid 2-related factor 2 (Keap1-Nrf2) inhibitors and their potential for targeting central nervous system diseases. J. Med. Chem..

[45] Xu W, Zhang N, Zhang Z, Jing P (2019). Effects of dietary cyanidin-3-diglucoside-5-glucoside complexes with rutin/Mg(II) against H2O2-induced cellular oxidative stress. Food Res. Int..

[46] Xiang XW (2019). Protective effect of seleno-amino-oligosaccharide on oxidative damage of IPEC-1 cells by activating Keap1/Nrf2 signaling pathway. Int. J. Biol. Macromol..

[47] Young ML, Franklin JL (2019). The mitochondria-targeted antioxidant MitoQ inhibits memory loss, neuropathology, and extends lifespan in aged 3xTg-AD mice. Mol. Cell Neurosci..

[48] Fuentes-Antras J (2015). Updating experimental models of diabetic cardiomyopathy. J. Diabetes Res..

[49] Khanam SS (2017). Prognostic value of short-term follow-up BNP in hospitalized patients with heart failure. BMC Cardiovasc. Disord..

