# Supplementary material for: Cyclovirobuxine D protects against diabetic cardiomyopathy by activating Nrf2-mediated antioxidant responses
Source: Sci Rep. 2020 Apr 14;10:6427. doi: 10.1038/s41598-020-63498-3 (PMC7156511; doi:10.1038/s41598-020-63498-3)
Supplement: Supplementary file 1 — Supporting information. [file 41598_2020_63498_MOESM1_ESM.pdf]

Cyclovirobuxine D protects against diabetic cardiomyopathy by activating Nrf2-mediated antioxidant responses

Zhaohui Jiang[1,2,3], Lingyun Fu[1,2,3],Yini Xu[1,2,3], Xiaoxia Hu[1,2,3], Hong Yang [1,2,3], Yanyan Zhang [1,2,3], Hong Luo [1,2,3] , Shiquan Gan[1,2,3], Ling Tao[1, 2], Guiyou Liang[1*], Xiangchun Shen[1,2,3,4 *]

*Correspondence: shenxiangchun@126.com and guiyou515@163.com

1. The State Key Laboratory of Functions and Applications of Medicinal Plants, School of Basic Medical Sciences, Guizhou Medical University, University Town, Guian New District, 550025,Guizhou, China.

2. The Department of Pharmacology of Materia Medica (The high efficacy application of natural medicinal resources engineering center of Guizhou Province and The high educational key laboratory of Guizhou province for natural medicianl Pharmacology and Druggability), School of Pharmaceutical Sciences, Guizhou Medical University, University Town, Guian New District, 550025,Guizhou, China.

3. The key laboratory of optimal Utilizaiton of Natural Medicine Resources (The union key laboratory of Guiyang City-Guizhou Medical Univeristy), School of Pharmaceutical Sciences, Guizhou Medical University, University Town, Guian New District, 550025,Guizhou, China.

4. The key laboratory of Endemic and Ethnic diseases（Guizhou Medical University），Ministry of Education, Guizhou Medical University, University Town, Guian New District, 550025,Guizhou, China.

SUPPLEMENTARY MATERIAL INVENTORY

SUPPLEMENTARY FIGURES

Supplementary Fig-S1. CVB-D ameliorates cardiac function in rats with DCM.

Supplementary Fig-S2. Effects of glucose at different concentrations on the viability of PNRCMs.

Supplementary Fig-S3. Effects of CVB-D at different concentrations on the viability of PNRCMs.

Supplementary Fig-S4. CVB-D promotes Nrf2 nuclear translocation *in vivo*.

Supplementary Fig-S5. Full-length western blots for Figures 2g.

Supplementary Fig-S6. Full-length western blots for Figures 3i.

Supplementary Fig-S7. Full-length western blots for Figures 4a.

Supplementary Fig-S8. Full-length western blots for Figures 4d.

Supplementary Fig-S9. Full-length western blots for Figures 4g.

Supplementary Fig-S10. Full-length western blots for Figures 4j.

Supplementary Fig-S11. Full-length western blots for Figures Figures5 b,d,f.

Supplementary Fig-S12. Full-length western blots for Figures 6e.

Supplementary Fig-S13. Full-length western blots for FigS-4.

SUPPLEMENTARY TABLES

Supplementary Table1. Effect of CVB-D on the binding free energy of Nrf2-keap1 complex

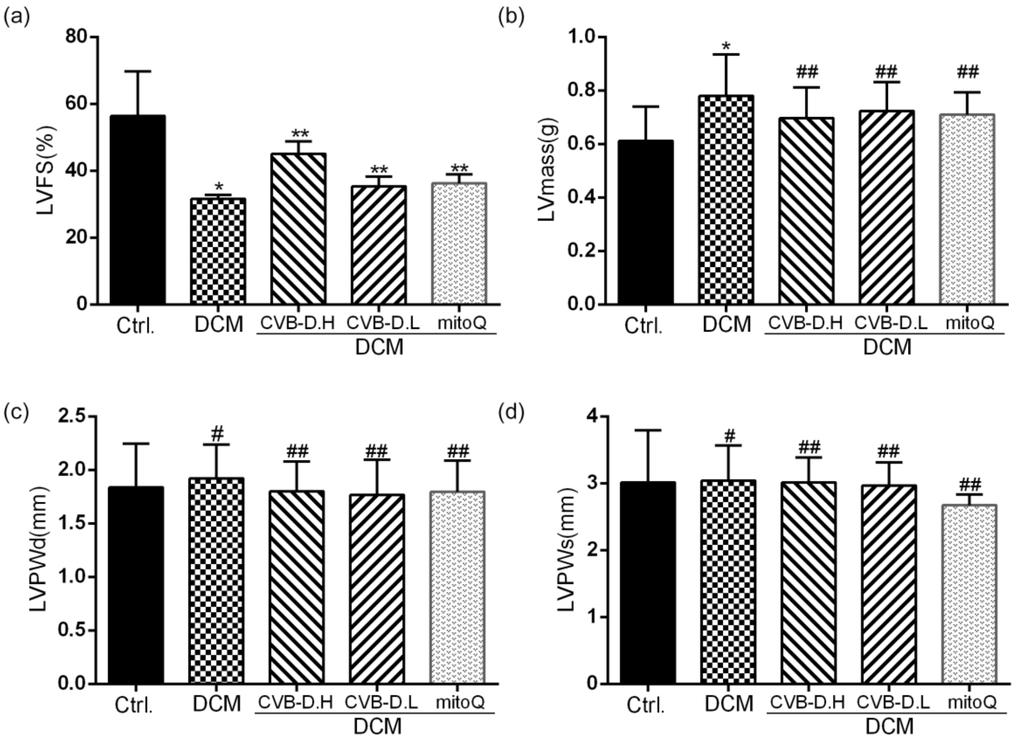

Supplementary Fig-S1. CVB-D ameliorates cardiac function in rats with DCM. Cardiac function parameters were indicated by (a) LVFS and (b) LVmass (c) LVPWd and（d）LVPWs. n（Ctrl.）=24; n（DCM）=7; n（DCM+CVB-D.H）=16; n（DCM+CVB-D.L）=8; n (DCM+mitoQ)=9. Data were expressed as the mean ±SD. *p < 0.05 versus the control group; #p > 0.05 versus the control group; **p < 0.05 versus the model group; ##p > 0.05 versus the model group.

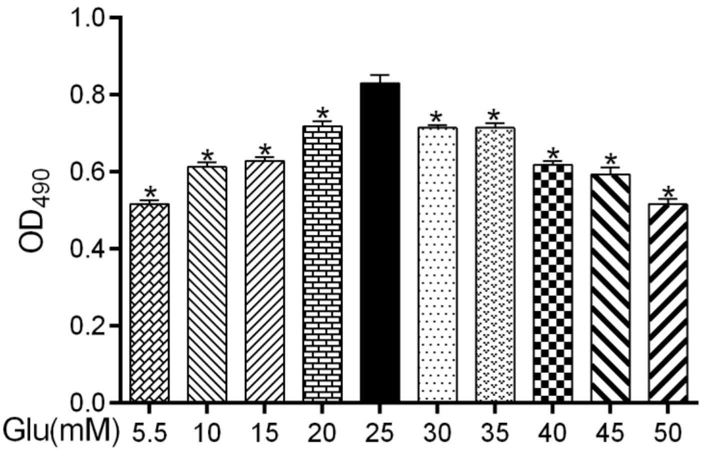

Supplementary Fig-S2. Effects of glucose at different concentrations on the viability of PNRCMs. PNRCMs were incubated in different concentrations of glucose for 72 hours,MTT measures cell viability. The data are presented as the mean ± SEM (n = 6 in each group), *$p < 0.05$ vs. the 25mM group.

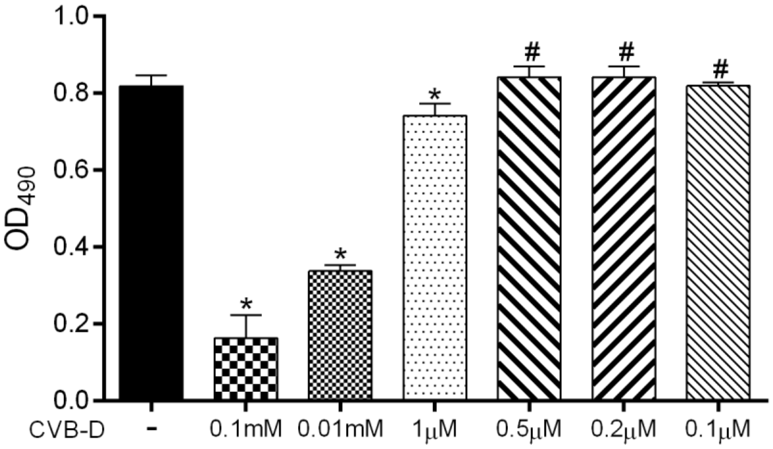

Supplementary Fig-S3. Effects of CVB-D at different concentrations on the viability of PNRCMs. PNRCMs were incubated in DMEM with 25 mM glucose for 72 hours, and then exposed to different concentrations of CVB-D for another 48 hours,MTT measures cell viability. The data are presented as the mean ± SEM (n = 6 in each group), *$p < 0.05$ vs. the control group; #$p > 0.05$ vs. the control group.

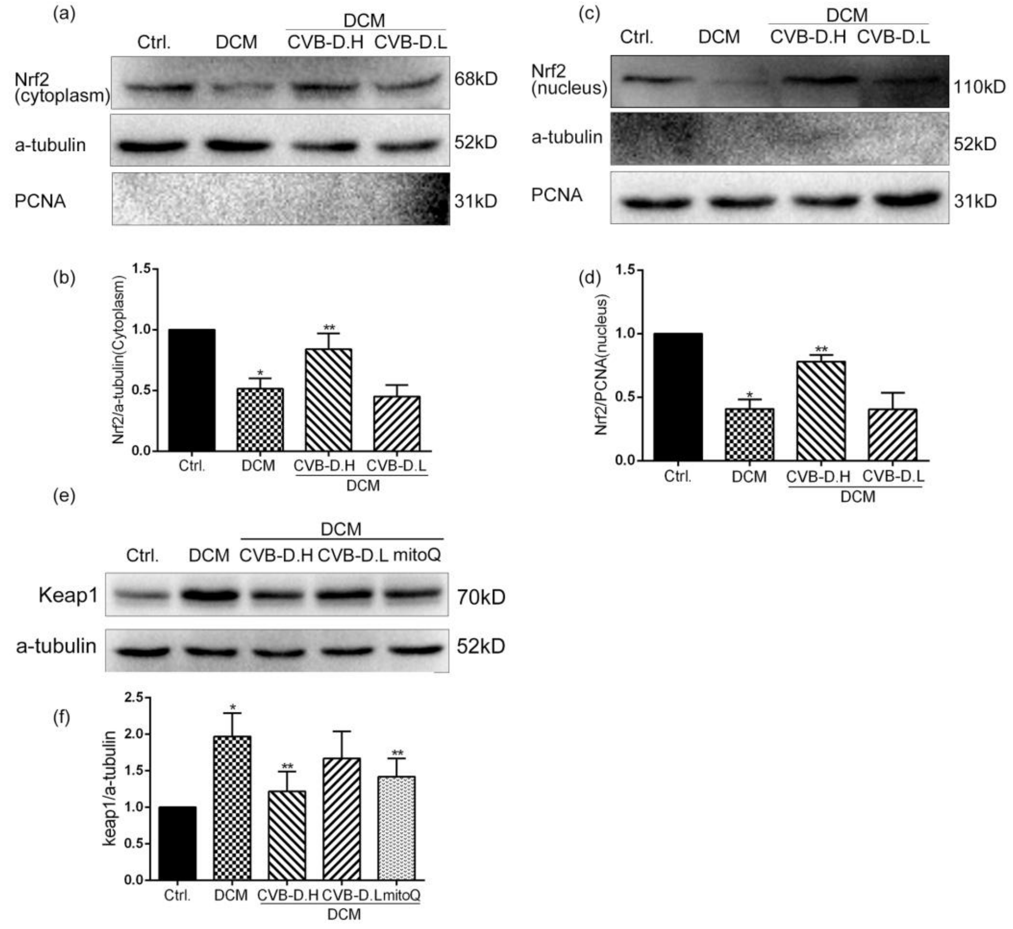

Supplementary Fig-S4. CVB-D promotes Nrf2 nuclear translocation *in vivo*. (a-d) The expression of cytoplasmic Nrf2 and nuclear Nrf2 in DCM heart were detected by western blotting. (e,f) The expression of Keap1 in DCM heart was assayed by western blotting analysis, full–length blots are presented in supplementary Fig-S13. The data are presented as the mean ± SEM (n = 3 in each group), *$p < 0.05$ vs. the control group; **$p < 0.05$ vs. the model group.

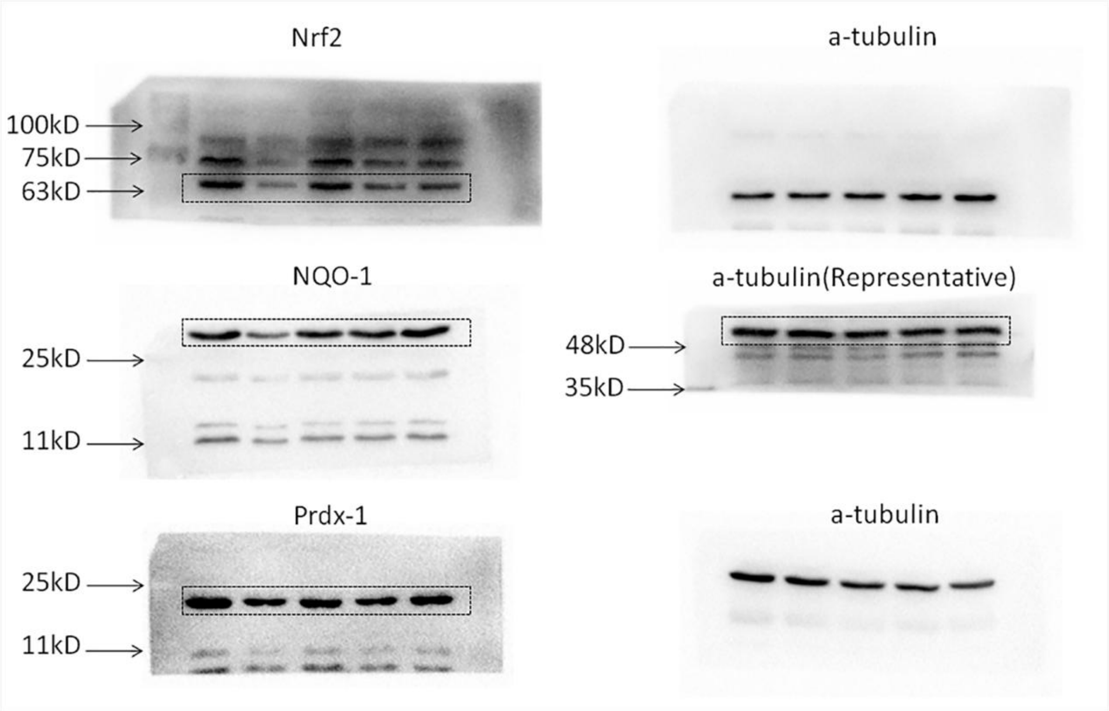

Supplementary Fig-S5. Full-length western blots for Figures 2g.

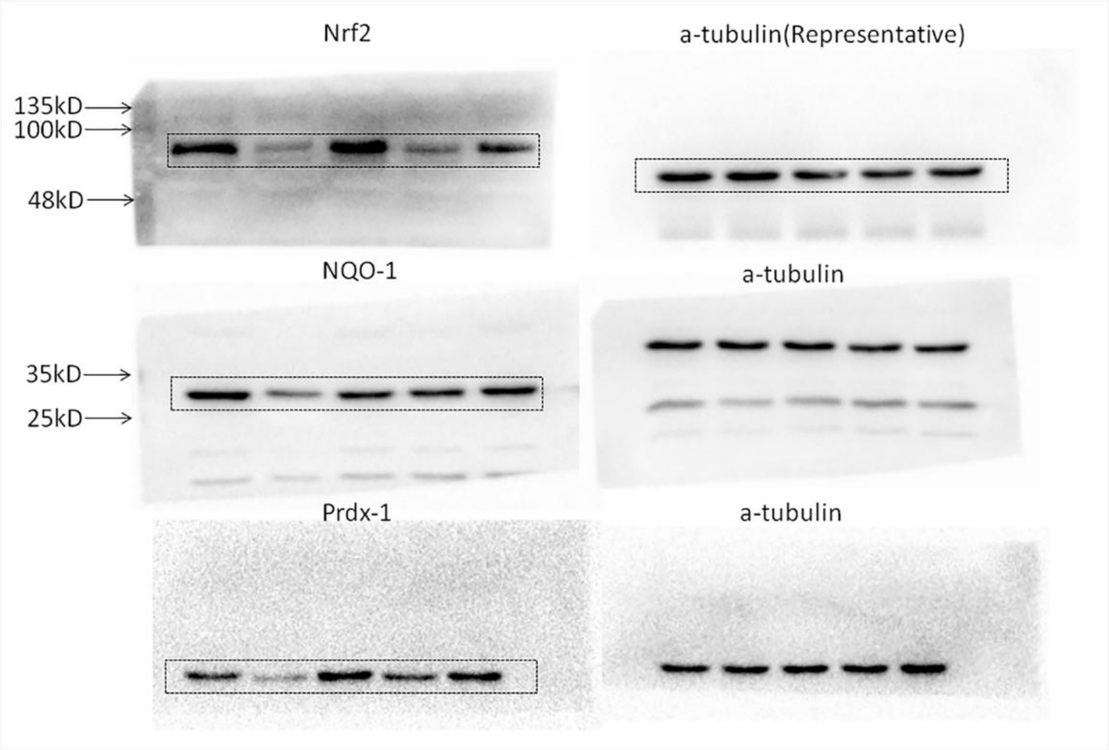

Supplementary Fig-S6. Full-length western blots for Figures 3i.

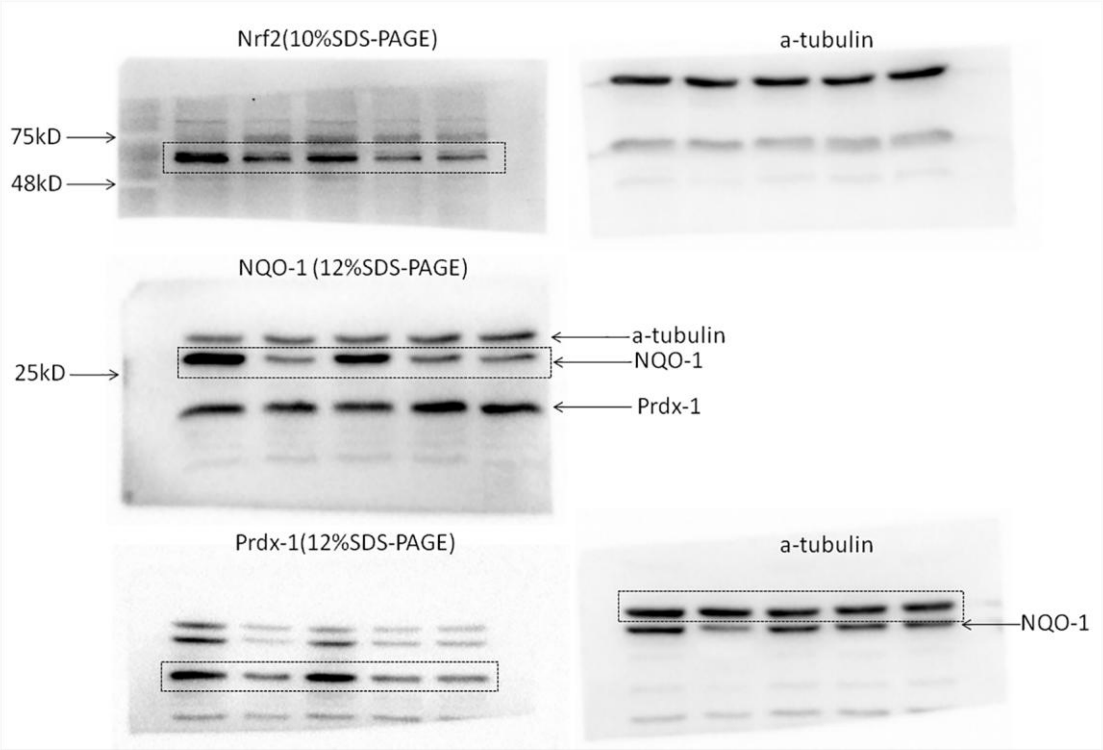

Supplementary Fig-S7. Full-length western blots for Figures 4a.

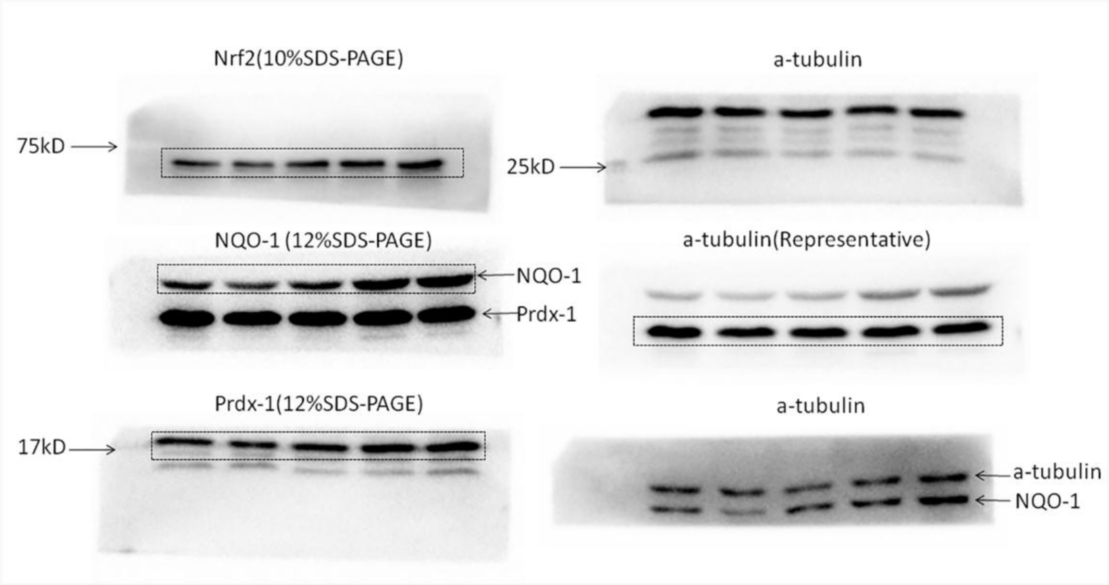

Supplementary Fig-S8. Full-length western blots for Figures 4d.

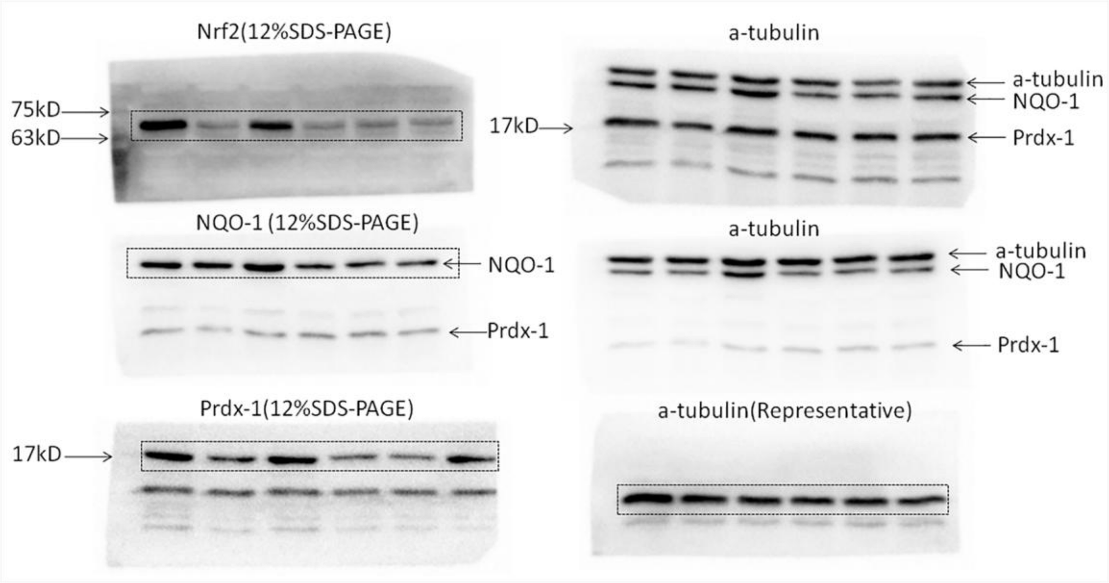

Supplementary Fig-S9. Full-length western blots for Figures 4g.

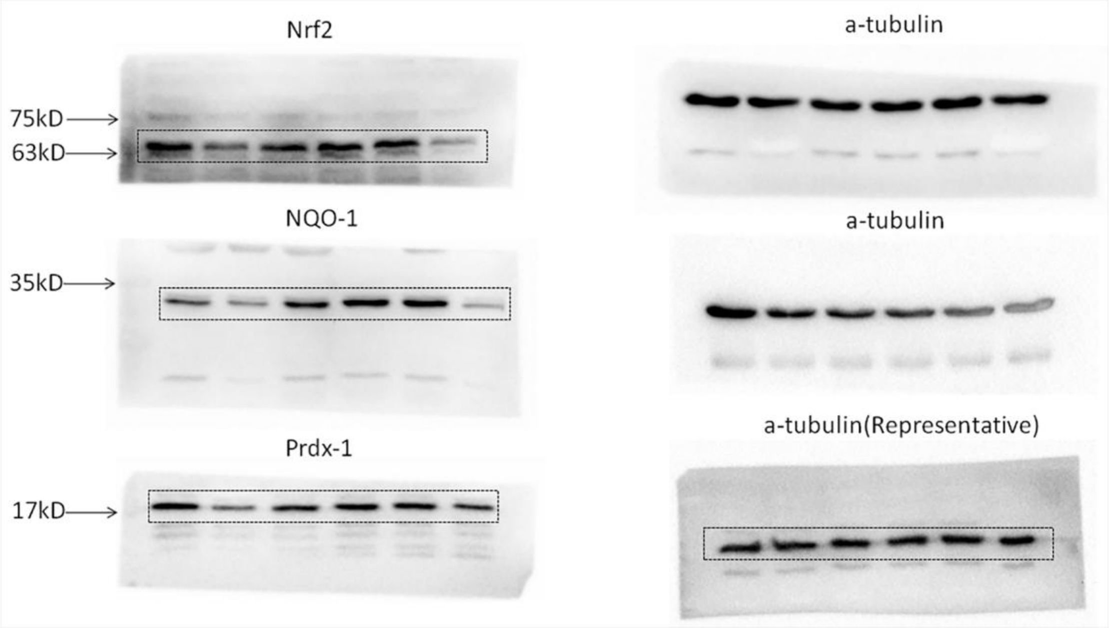

Supplementary Fig-S10. Full-length western blots for Figures 4j.

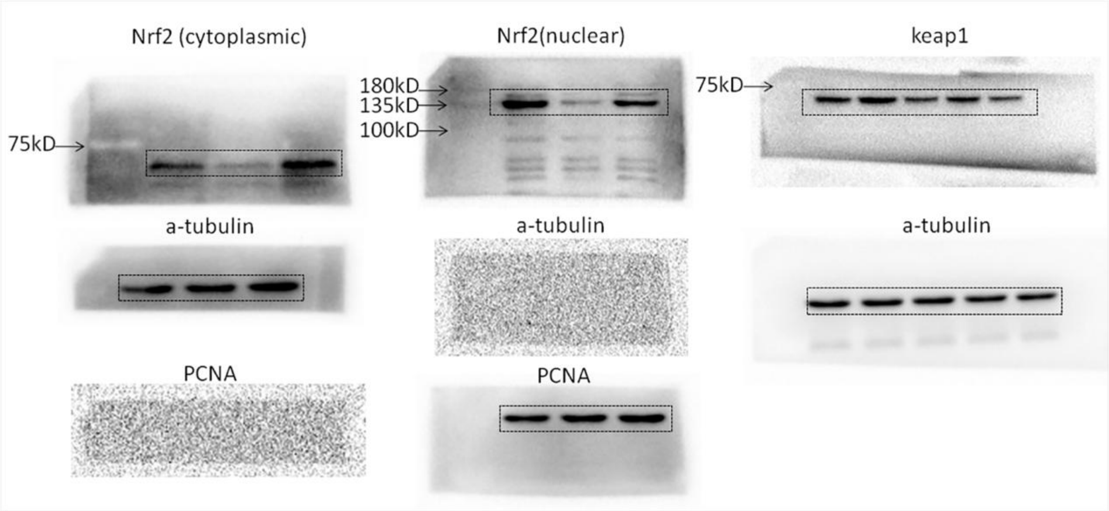

Supplementary Fig-S11. Full-length western blots for Figures5 b,d,f.

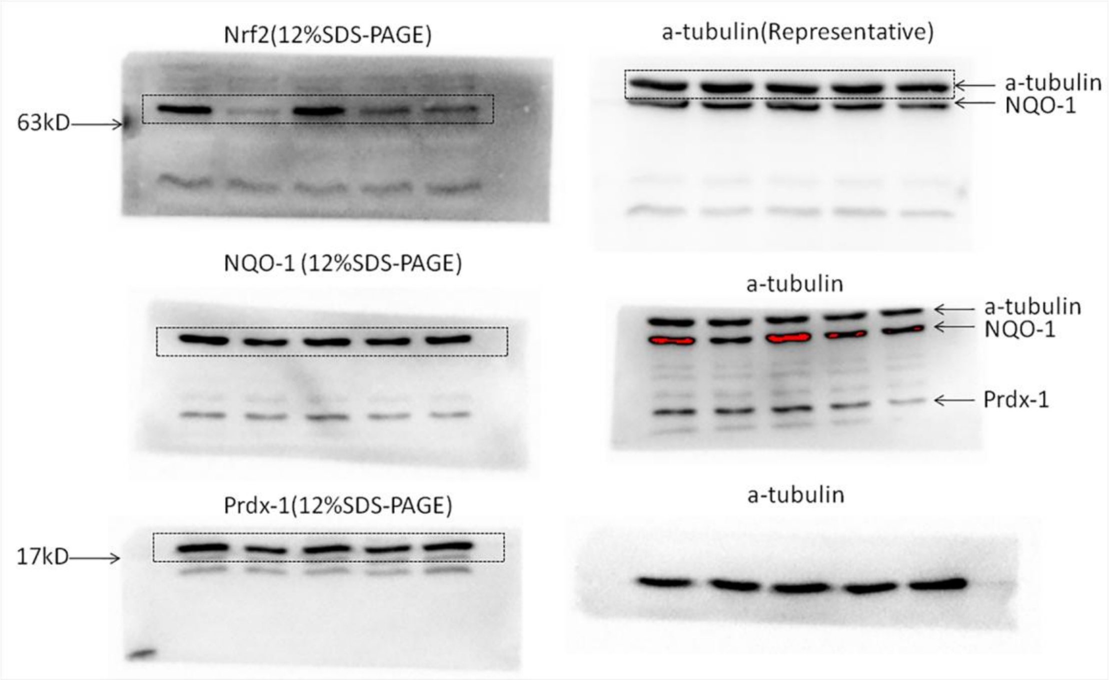

Supplementary Fig-S12. Full-length western blots for Figures6e.

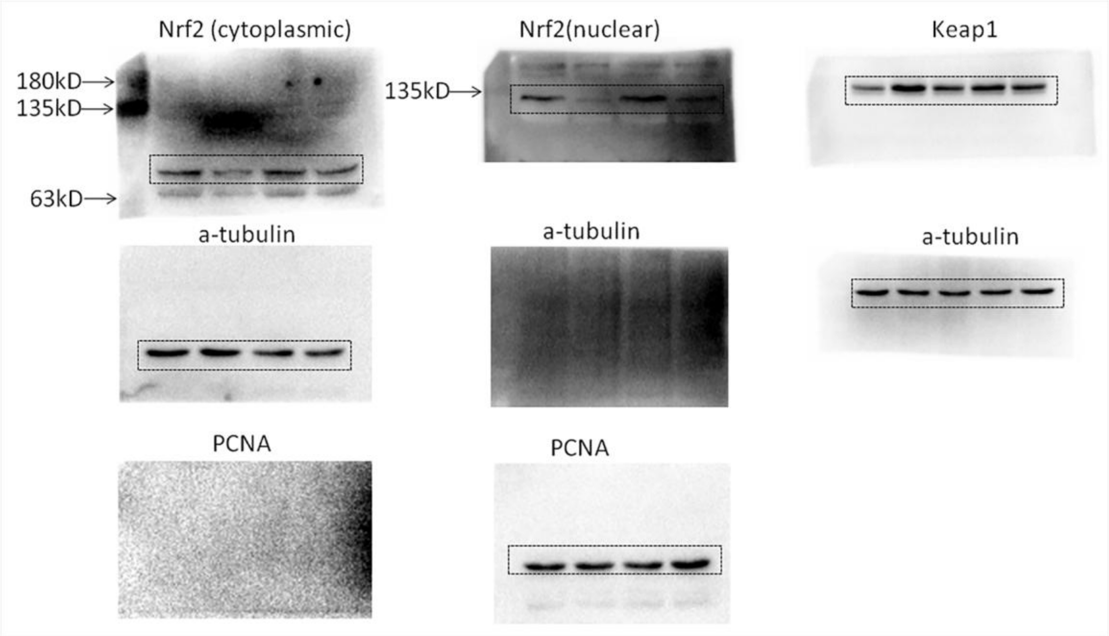

Supplementary Fig-S13. Full-length western blots for FigS-4.

Supplementary Table1. Effect of CVB-D on the binding free energy of Nrf2-keap1 complex

	**Nrf2-keap1**	**Nrf2-keap1-CVB-D**
**VDWAALS(**kcal/mol**)**	-56.6±4.8	-55.4±7.7
**EEL(**kcal/mol**)**	-339.4±44.5	-318.9±43.9
**EGB(**kcal/mol**)**	339.4±39.2	327.7±36.0
**ESURF(**kcal/mol**)**	-9.8±0.5	-10.0±0.7
**△G gas(**kcal/mol**)**	-396.0±43.3	-374.3±41.9
**△G sol(**kcal/mol**)**	329.6±39.0	317.6±36.2
**△G $_{bind}$(**kcal/mol**)**	-66.4±7.1	-56.7±8.3**

VDWAALS，van der Waals energy；EEL, electrostatic energy；EGB，polarized solvation energy eased on Generalized Bonn model；ESURF, nonpolar solvation energy calculated based on solvent accessible surface area. △G gas, calculated gas phase energy; △G sol, calculated solvation energy; △G $_{bind}$, calculated binding free energy. △G gas= VDWAALS+ EEL； △G $_{sol}$= EGB+ ESURF； △G $_{bind=}$△G $_{gas}$+△G $_{sol}$. ***p*=0.196 vs. Nrf2-keap1.
